# The Cyanobacterial Ribosomal-Associated Protein LrtA from *Synechocystis* sp. PCC 6803 Is an Oligomeric Protein in Solution with Chameleonic Sequence Properties

**DOI:** 10.3390/ijms19071857

**Published:** 2018-06-24

**Authors:** Lellys M. Contreras, Paz Sevilla, Ana Cámara-Artigas, José G. Hernández-Cifre, Bruno Rizzuti, Francisco J. Florencio, María Isabel Muro-Pastor, José García de la Torre, José L. Neira

**Affiliations:** 1 Center for Environmental Biology and Chemistry Research, Facultad Experimental de Ciencias y Tecnología, Universidad de Carabobo, 2001 Valencia, Venezuela; lellyscontreras@gmail.com; 2 Facultad de Farmacia, Departamento de Química Física II, Universidad Complutense de Madrid, 28040 Madrid, Spain; paz@farm.ucm.es; 3 Instituto de Estructura de la Materia, IEM-CSIC, Serrano 121, 28006 Madrid, Spain; 4 Department of Chemistry and Physics, Research Centre CIAIMBITAL, University of Almería- ceiA3, 04120 Almería, Spain; acamara@ual.es; 5 Department of Physical Chemistry, University of Murcia, 30003 Murcia, Spain; jghc@um.es (J.G.H.-C.); jgt@um.es (J.G.d.l.T.); 6 CNR-NANOTEC, Licryl-UOS Cosenza and CEMIF.Cal, Department of Physics, University of Calabria, 87036 Rende, Italy; 7 Instituto de Bioquímica Vegetal y Fotosíntesis, CSIC-Universidad de Sevilla, 41092 Seville, Spain; floren@us.es (F.J.F.); imuro@ibvf.csic.es (M.I.M.-P.); 8 Instituto de Biología Molecular y Celular, Universidad Miguel Hernández, 03202 Elche (Alicante), Spain; 9 Instituto de Biocomputación y Física de Sistemas Complejos, Joint Units IQFR-CSIC-BIFI, and GBsC-CSIC-BIFI, Universidad de Zaragoza, 50009 Zaragoza, Spain

**Keywords:** conformational plasticity, disordered protein, folding, ribosomal protein, spectroscopy, protein stability

## Abstract

The LrtA protein of *Synechocystis* sp. PCC 6803 intervenes in cyanobacterial post-stress survival and in stabilizing 70S ribosomal particles. It belongs to the hibernating promoting factor (HPF) family of proteins, involved in protein synthesis. In this work, we studied the conformational preferences and stability of isolated LrtA in solution. At physiological conditions, as shown by hydrodynamic techniques, LrtA was involved in a self-association equilibrium. As indicated by Nuclear Magnetic Resonance (NMR), circular dichroism (CD) and fluorescence, the protein acquired a folded, native-like conformation between pH 6.0 and 9.0. However, that conformation was not very stable, as suggested by thermal and chemical denaturations followed by CD and fluorescence. Theoretical studies of its highly-charged sequence suggest that LrtA had a Janus sequence, with a context-dependent fold. Our modelling and molecular dynamics (MD) simulations indicate that the protein adopted the same fold observed in other members of the HPF family (β-α-β-β-β-α) at its N-terminal region (residues 1–100), whereas the C terminus (residues 100–197) appeared disordered and collapsed, supporting the overall percentage of overall secondary structure obtained by CD deconvolution. Then, LrtA has a chameleonic sequence and it is the first member of the HPF family involved in a self-association equilibrium, when isolated in solution.

## 1. Introduction

The *lrtA* gene was first identified in *Synechococcus* sp. PCC 7002 as a sequence encoding a light-repressed protein [1], with a larger half-life in dark conditions than in the presence of light [2]. Although the exact functions of the LrtA protein are unknown, recent studies have shown that it is involved in post-stress survival in *Synechocystis* sp. PCC 6803, stabilizing the 70S ribosomal particles [3].

LrtA is related to other proteins, which are highly present among bacteria and associated with ribosomes. These proteins modulate ribosome activity to preserve their integrity and aid in cell survival during stress circumstances. Under these conditions, stalling of the protein synthesis, a major energy-consuming process in living cells, is downregulated, usually by proteins involved in ribosome inhibition. Reduction of translation activity is associated with: (i) dimerization of 70S particles to form the translationally inactive 100S disome (also known as hibernating ribosomes [4]), mediated by intermolecular interactions among proteins; or alternatively; (ii) interaction of canonical ribosomal proteins with the ribosome [5,6]. Among the most studied members of this protein family are two *Escherichia coli* proteins: YfiA (also known as PY or RaiA, ribosome associated inhibitor A); and YhbH (also known as HPF, hibernation promoting factor). YfiA is thought to inhibit translation indirectly, by modulating a more stringent proofreading mechanism involving 70S particles [7,8]. On the other hand, HPF stops translation by stabilizing 100S dimers [8,9,10]. Formation of 100S disomes is also mediated by other proteins, known as ribosome modulation factors (RMFs), in γ-proteobacteria species or double YfiA- and YhbH- knocked-out cells [10]. Phylogenetic analyses have shown that most bacteria have at least one of those HPF or YfiA homologues [10]. These homologues have been classified in three classes, based on the presence of a conserved domain and, in some cases, additional sequence extensions: long HPF, short HPF, and YFiA. The conserved domain has a β-α-β-β-β-α fold, with the two α-helices packed against one side of the four-stranded β-sheet [5,11]. According to its sequence, the LrtA from *Synechocystis* sp. PCC 6803 could be classified within the long HPF sub-family; in addition, it also bears similarity to the spinach plastid-specific ribosomal protein, which is present in the chloroplast stroma, either associated or unbound to the 30S ribosomal unit [3]. Although we have shown that LrtA stabilizes 70S particles [3], nothing is known about the conformation or stability of the isolated protein in solution.

In this work, we embarked in the characterization of the conformational stability and structure of LrtA from *Synechocystis* sp. PCC 6803 by using experimental and *in silico* approaches. Our results are the first characterization of the conformation and stability of a member of the long HPF subfamily. At physiological pH, LrtA was involved in a self-association equilibrium, as shown by hydrodynamic techniques. The protein acquired a native-like conformation around pH 6.0, as judged from intrinsic fluorescence (monitoring tertiary structure), ANS (8-anilino-1-naphthalene sulfonic acid) fluorescence (revealing hydrophobic solvent-exposed patches), CD (reporting secondary structure) and 1D ^1^H NMR experiments. The MD simulations and analyses of sequence suggested that the protein had a Janus sequence, and its conformation was solvent-dependent, with an N-terminal region acquiring the fold of other members of the HPF family and the C-terminal region appearing disordered and collapsed. Therefore, LrtA is the first member of the HPF family with chameleonic features encoded in its sequence and shown to be involved in a self-association equilibrium when isolated in solution.

## 2. Results

### 2.1. Isolated LrtA Was Involved in a Self-Association Equilibrium in Solution

We first tried to elucidate the oligomerization state of the protein to identify the protein-concentration range where we must characterize the conformational stability of the protein.

To map the hydrodynamic properties of LrtA we used three hydrodynamic techniques: DOSY-NMR (diffusion ordered NMR spectroscopy), DLS (dynamic light scattering), size exclusion chromatography (SEC), glutaraldehyde cross-linking, and lifetime fluorescence measurements. Furthermore, we tried to measure the self-association of LrtA by using isothermal titration calorimetry (ITC), but in all attempts, protein precipitated at the concentrations required to carry out the experiments. It is important to pinpoint the differences among the different hydrodynamic techniques used in this work. With NMR, we shall obtain information about the low-molecular weight species, whose overall rotational tumbling is very fast. By using DLS, we shall obtain information about the hydrodynamic parameters, assuming a spherical shape, for all the species (high or low molecular weight) present in solution, and we shall be able to see whether those hydrodynamic parameters are protein-concentration-dependent. By using SEC, we shall be able to monitor the elution volume of LrtA, which will depend on the molecular weight and the shape of the molecule, but that volume could be also affected by possible interactions with the column. Finally, by lifetime fluorescence measurements, we shall determine how the decay of the electronic excited states can be affected by: (i) the presence of conformational isomers; (ii) energy transfer among the eight Tyr residues in LrtA; or (iii) even transient electronic effects in collisional quenching [12,13]. To elucidate the oligomerization state of the protein, we also tried to carry out *T*_2_ echo measurements estimating the averaged correlation time of the species present in solution from the amide region. However, at the times used (2.9 ms and 400 μs) in our echo experiments, most of the amide peaks disappeared, and only the proton resonances of the His ring (around 8.5 ppm) could be clearly measured, yielding a very long value for the *T*_2_, unreliable to estimate the mobility of the backbone of the polypeptide chain.

The DOSY-NMR measurements at pH 8.0 yielded a translational diffusion coefficient (*D*) with a value of (7.2 ± 0.2) × 10^−7^ cm^2^ s^−1^ (Figure S1A). By taking into account the hydrodynamic radius, *R*_S_, of dioxane (2.12 Å), and its *D* under our conditions ((6.8 ± 0.2) × 10^−6^ cm^2^ s^−1^), the *R*_S_ (Stokes radius) estimated for LrtA was 20 ± 2 Å. We can compare this value with that theoretically determined for a polypeptide with the length of LrtA. The *R* value for an unsolvated, ideal, spherical molecule can be estimated from [14]: $R=\sqrt[{3}]{3M\bar{V}/4N_{A}\pi }$, where *N_A_* is Avogadro’s number, *M* is the molecular weight (22.717 kDa) and $\bar{V}$ the specific volume of LrtA (0.729 mL/g). The calculated *R* for LrtA is 18.7 Å, but since the hydration shell is 3.2 Å wide [15], the hydration radius would be 21.9 Å, which is similar to the *R*_S_ from DOSY-NMR. On the other hand, it has been shown that the *R*_S_ of a folded spherical protein can be approximated by [16]: $R_{S}=(4.75\pm 1.11)N^{0.29}$, where *N* is the number of residues; in a 197-residue-long protein such as LrtA, this expression yields 21 ± 6 Å, similar to that determined by DOSY-NMR experiments. Therefore, by the DOSY-NMR measurements, we are only detecting a monomeric globular species of LrtA. In fact, the 1D ^1^H-NMR spectrum of LrtA at pH 8.0 (with 500 mM NaCl) (Figure S2) corresponds to that of a well-folded protein with dispersed peaks in the methyl and amide regions; interestingly enough, the spectrum had down-field shifted H_α_ protons (between 5.0 and 6.0 ppm), suggesting the presence of residues involved in β-strands. It is interesting to note that, as possible higher-order molecular species could not be observed in the NMR spectrum due to their molecular weight (and therefore signal broadening), the presence of the majority of the amide protons belonging to possible disordered regions in the protein (see below, 2.4.) would not be observed in the amide region due to: (i) solvent hydrogen-exchange at pH 8.0; (ii) conformational exchange broadening; or (iii) overlapping with the signals from the well-folded region (as indicated by the largest increase of intensity around 8.3 ppm, Figure S2B). In addition, the alkyl resonances belonging to possible polypeptide disordered regions would be hindered by the rest of the methyl groups of the protein in the up-field shifted region. On the other hand, the 1D ^1^H-NMR spectrum at pH 4.5 showed a smaller intensity and a poorer signal-to-noise ratio (due probably to the precipitation during sample preparation) than the spectrum at pH 8.0. In addition, the spectrum also showed broader peaks (and less intense, as it is evident by comparing Figure S3B with Figure S2B), and the absence of well-dispersed signals in the amide and methyl regions (Figure S3), as shown, for instance, by the lack of peaks around 0.3 and 0.5 ppm, which appeared at pH 8.0. The broader peaks at pH 4.5 (when compared to pH 8.0) could be due to the presence of uni-molecular conformational-exchange equilibria or, alternatively, to the presence of self-associated species. Thus, to elucidate whether at pH 4.5 there were concentration-dependent equilibria, we carried out far UV CD experiments at protein concentrations of 4.5 and 9.8 μM (in protomer units); these experiments (Figure S4) showed that the molar ellipticity and the shape of the spectra were protein-concentration-dependent. Therefore, these results suggest that the conformation of the protein and its self-associated features were different at the two pH values of 4.5 and 8.0.

**Figure 1:**
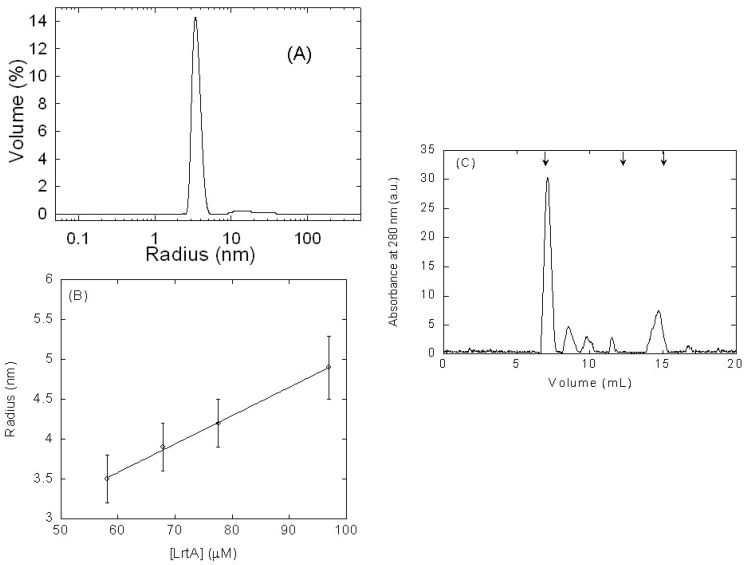
Hydrodynamic measurements of LrtA by dynamic light scattering (DLS) and size exclusion chromatography (SEC): (**A**) DLS measurements of the hydrodynamic radius RS of LrtA as a function of the percentage of the volume peak at 68 μM concentration (in protomer units). (**B**) Variation of the calculated *R*_S_ with LrtA concentration (in protomer units). Error bars are standard deviations from the fitting to a spherical shape. (**C**) SEC chromatogram of LrtA at 97 μM (in protomer units) at pH 8.0 (50 mM Tris) in 0.7 M NaCl; the arrows at the top indicate (from left to right) the elution volumes of blue dextran (7.1 ± 0.1 mL), albumin (12.1 ± 0.1 mL; 63.7 kDa), and bovine RNase A (15.1 ± 0.1 mL; 15.7 kDa) (the errors are standard deviations of three independent measurements). Chromatogram was baseline-corrected by UNICORN 5.01 software (GE Healthcare), and therefore, the origin of the sharpening observed in the peaks. Experiments were carried out at 25 °C.

Second, we measured the hydrodynamic features of LrtA by using DLS at several protein concentrations. Two peaks were identified in the size distribution analysis for the concentration of 68 μM (in protomer units): the first peak with *R*_S_ = 39 ± 5 Å that accounts for the 97.5% of the protein in the solution and a second peak with *R*_S_ = 409 ± 200 Å corresponding to a small amount of aggregates (Figure 1A). Taking into account that particle scattering intensity is proportional to the square of the molecular weight, a small percentage (in this case 2.5%) of protein aggregates dominates the intensity distribution, which can be misleading, and therefore, the results in Figure 1A are shown as size distribution by volume instead of intensity. That *R*_S_, obtained from the first peak, corresponds to a molecular weight of 81 kDa, by using an empirical mass vs. size calibration curve in the instrument software. The experiments at different LrtA concentrations indicate that the *R*_S_ (obtained from the volume peak measurements of the first observed peak) varied with the protein concentration. These findings suggest the presence of a self-associated equilibrium at pH 8.0 (Figure 1B) involving the protein, as also described in other oligomeric proteins [17]. It is interesting to note that these oligomeric species should not be observed in NMR due to their large molecular weights [15].

Third, we also detected the presence of oligomeric species using glutaraldehyde cross-linking, which can react as monomer, but also as a heterogeneous polymer, involving accessible lysine residues. Our results (Figure S5) indicate that in the presence of a final concentration of 1% gutaraldehyde, there were dimers (which appear close to the band of the protein marker at 48 kDa) and other high-molecular-weight species with molecular weights larger than 210 kDa, at the top of the SDS-PAGE (sodium dodecyl sulfate polyacrylamide gel electrophoresis) gel lanes. These high molecular weight species could be due to the presence of cross-linked dimeric species.

Next, we used SEC in a Superose 12 10/300 GL, in buffer pH 8.0 (50 mM Tris) with 0.7 M NaCl to elucidate whether the protein behaved as an oligomer. We used such high concentrations of NaCl to avoid, as much as possible, any kind of protein-column interactions (as those we have observed to occur with other kind of matrix columns, see below Section 4). The protein markers used to calibrate the column were also loaded in the same buffer. At loading concentrations of 97 μM (in protomer units) of LrtA, the protein eluted as several peaks (errors are standard deviations from three independent measurements) at 7.3 ± 0.2, 8.5 ± 0.1, 9.8 ± 0.2, 11.5 ± 0.1, and 14.7 ± 0.2 mL (Figure 1C). These peaks, especially that at 7.3 mL, indicate that the protein behaved as an oligomer, with molecular weights larger than those of albumin (63.7 kDa) and bovine RNase A (15.7 kDa), although other higher-order molecular species of LrtA were present in solution. The peak at 14.7 mL could be due to the monomeric species, which was observed under these conditions. The other oligomeric species could be assigned to hexamers (11.5 mL) and dodecamers (9.8 mL), whereas the other two could be due to the presence of aggregates (as those species detected in DLS, see above); however, it is important to indicate that some of the peaks could be also due to protein-column interactions even in the presence of high NaCl concentration.

Finally, we measured the fluorescence lifetimes of LrtA at different protein concentrations. The experimental decay of the total protein fluorescence was best fit to bi-exponential functions (Table 1, Figure S6), and thus, two lifetimes were observed; attempts to fit the experimental data to more than two exponentials led to an increase in the χ^2^. At any of the protein concentrations, the shortest lifetime corresponded to the largest amplitude (a_1_), and it did not change with the protein concentration. Interestingly enough, the longest lifetime (as well as its amplitude, a_2_) was concentration-dependent (Table 1). Furthermore, the <τ> showed also a concentration-dependence: going from a value of 6 ns (at the smallest concentration) to 1 ns at 98 μM. It is well-known that the intrinsic fluorescence lifetime of the first excited electronic singlet state does not change, but due to various quenching processes, changes in the environment around the fluorophores or even conformational changes in the molecular species, the measured lifetime is different from the intrinsic one [12,13]. Thus, even ruling out a possible fitting of the data to a monomer ↔ oligomer equilibria because we are observing the lifetimes of eight Tyr residues, each of them with a different environment, we can conclude that the concentration-dependence observed in the <τ> (Table 1) should have its origin in association-dissociation events.

It could be thought that the detected self-associated species could be random-oligomers; although we cannot rule out the presence of aggregates (from the DLS, SEC, and glutaraldehyde cross-linking results), however, there are at least three pieces of evidence suggesting that the self-associated species do not oligomerize un-specifically: (i) the DLS results show a linear dependence with the concentration (Figure 1B); (ii) the protein-dependent, and almost exponential, variation of the life-times; and, (iii) the presence of bands at particular molecular weights in the glutaraldehyde experiments. Therefore, those results, together with experiments from (GdmCl) chemical denaturations (see below, Section 2.3.), must be due to the presence of self-associated equilibria, involving the regions around some of the eight Tyr residues.

### 2.2. LrtA Acquired a Native-Like Conformation Between pH 6.0 and 9.0

We analyzed the structure of LrtA at varying pH to find out in which interval the protein acquired a native-like conformation. To this end, we used several biophysical techniques, namely, intrinsic and ANS fluorescence, CD and NMR. We used intrinsic fluorescence to monitor changes in the tertiary structure around its eight Tyr residues. Furthermore, ANS fluorescence was used to monitor the burial of solvent-exposed hydrophobic patches. We acquired far-UV CD spectra to monitor the changes in secondary structure. Finally, we acquired 1D ^1^H NMR spectra that show the presence of secondary and tertiary structure at physiological pH (see above, Section 2.1.). These spectra indicate (Figures S2 and S3) that the secondary and tertiary structures of the protein at pH 4.5 and 8.0 were completely different.

#### 2.2.1. Fluorescence

**Figure 2:**
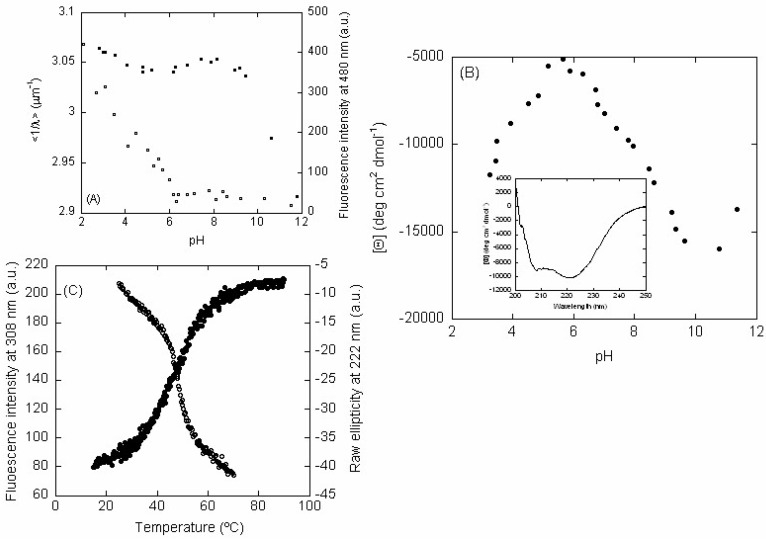
pH-denaturation of LrtA: (**A**) Intrinsic (left axis, filled circles) and ANS (right axis, blank squares) fluorescence of LrtA, as the pH was modified. (**B**) Changes in the [⊝] at 222 nm as the pH was varied (filled circles). Inset: far-UV CD spectrum of LrtA at 100 mM phosphate buffer (pH 8.0), with 500 μM NaCl at 25 °C. (**C**) Thermal denaturations followed by intrinsic fluorescence (left axis, blank circles) at pH 7.0 and 5 μM (in protomer units) of LrtA, and raw ellipticity at 222 nm at pH 7.0 and 9.8 μM, in protomer units (right axis, filled circles).

Intrinsic Steady-State Fluorescence and Thermal Denaturations—The fluorescence spectrum of LrtA at physiological pH showed a maximum at 308 nm, as expected for a polypeptide chain with fluorescent Tyr residues. The pH-dependence of the intrinsic <1/λ> showed two transitions (Figure 2A, left axis, filled circles). The first transition finished at pH 6.0, but we could not determine its p*K*_a_ due to the absence of an acidic baseline. This transition was probably due to the titration of some of the seventeen Glu and/or twelve Asp residues of the LrtA sequence [18,19], which can alter the environment around some of the Tyr residues. However, we cannot rule out that it could be also due to the titration of some of the eight naturally-occurring His residues, taking place at an unusually low p*K*_a_. The second transition occurred at basic pH, starting at pH > 9.0, but, in this case, we could not determine the p*K*_a_ due to the absence of a baseline at the highest pH values. This transition was probably due to the titration of at least some of the eight Tyr residues in the sequence. Therefore, the changes observed in fluorescence as the pH was changed could be due to titrations of specific residues around the Tyr residues, or alternatively, to conformational changes involving those fluorescent amino acids.

Thermal denaturations of LrtA were carried out at several pH values with a protein concentration of 9.8 μM, in protomer units. At pH values larger than 6.0, we observed an irreversible broad transition (Figure 2C, left axis, blank circles). Below pH 6.0, we did not observe any sigmoidal behavior, and we did not observe any sigmoidal transition at pH 13.0 either (Figure S7A). It could be argued that as fluorescence is intrinsically temperature-sensitive [12,13], we are not monitoring the denaturation of the protein. However, it must be kept in mind that fluorescence temperature sensitivity is linear (as observed at low pH values, Figure S7A; or in the native and unfolded baselines of the curve shown in Figure 2C), but it is not sigmoidal as observed in the denaturations at pH 7.0, with a midpoint around 45 °C (Figure 2C), or at pH 8.4 (Figure S7A). We also carried out experiments at LrtA concentrations of 5 μM, in protomer units (Figure 2C), and denaturation was also irreversible. Therefore, irreversibility was not associated with the amount of protein used during thermal denaturations.

ANS-Binding—At low pH, the ANS fluorescence intensity at 480 nm was large and decreased as the pH was raised (Figure 2A, right axis, blank squares), suggesting that LrtA had solvent-exposed hydrophobic regions. We could not determine the p*K*_a_ of this titration due to the absence of an acidic baseline. The burial of solvent-exposed hydrophobic residues was complete at pH 6.0, as it happens with the transition observed by following the intrinsic fluorescence (see above). Since ANS reports on burial of hydrophobic surface, and therefore it monitors conformational changes, we must conclude that the protein had structural changes at acidic pH values; then, the variations monitored by intrinsic fluorescence (see above) at acidic pH must be associated with conformational changes due to the protonation of Asp and Glu residues (or the other amino acids described above).

In conclusion, our results indicate that, at low pH values, LrtA had solvent-exposed hydrophobic regions.

Solvent-Exposure of Tyr Residues Monitored by Iodide and Acrylamide Quenching—We carried out quenching experiments at pH 3.0, 7.0, and 11.0, because these are the three regions where we observed a different intrinsic fluorescence behavior of LrtA (Figure 2A). We used two quenching agents because of the charge effects probably occurring at extreme pH value with I^−^. We have assumed that the fluorescence lifetimes of the self-associated protein (for a fixed protein concentration) did not change in the whole pH interval. The *K*_sv_ values for KI and acrylamide in the absence of denaturant were smaller than those measured in other proteins containing only Tyr residues [20,21] (Table 2). As a general trend, the *K*_sv_ values of LrtA in the presence of acrylamide were smaller at acidic pH values than at physiological or basic ones; these differences could be due to the presence of higher-order self-associated species at the acidic pH values (as suggested by the ANS results (Figure 2A) and the CD data at low pH (Figure S4)). Furthermore, these results indicate that the structure of LrtA underwent some conformational changes at acidic pH (in agreement with results from intrinsic and ANS fluorescence, Figure 2A, and the NMR results, Figures S2 and S3). In the presence of GdmCl (guanidine hydrochloride), the *K*_sv_ values were larger (either in KI or acrylamide) than those in the absence of denaturant (Table 2), suggesting that Tyr residues were more solvent-exposed.

#### 2.2.2. CD

The far-UV (ultraviolet) CD spectrum of LrtA at pH 8.0 had minima at 222 nm and 210 nm (Figure 2B inset), suggesting the presence of helix- or turn-like conformations. Decomposition of the far-UV CD spectrum at pH 8.0, by using the k2d algorithm, available online at the DICHROWEB site [22,23], yields a 30% of helical structure, 17% of β-sheet and 52% of random-coil. However, as LrtA has 8 Tyr, 4 Phe, and 14 His residues (eight naturally-occurring residues and six in the purification tail, see Section 4), we cannot rule out the absorbance of aromatic residues at this wavelength [24,25].

The molar ellipticity, [⊝], at 222 nm showed a dumb-bell shape, with a maximum value at pH 6.0 (Figure 2B). These results suggest that there were changes in the secondary structure (or alternatively in the environment around aromatic residues [24,25]) above and below pH 6.0; interestingly enough, the changes at low pH mirrored those observed by intrinsic and ANS fluorescence (Figure 2A). Since the fluorescence results indicated that the environment around Tyr residues remained essentially unaltered until pH 9.0 (Figure 2A, filled circles), and taking into account the ANS-fluorescence (Figure 2A, blank circles), we can conclude that between pH 6.0 and 9.0, although the protein had a folded conformation (Figure S2A,B), either the secondary structure of the protein changed or, alternatively, the environment around some of the 4 Phe and 14 His residues in LrtA. We could not determine the p*K*_a_ values corresponding to the titrations at the two sides of the curve due to the absence of acidic and basic baselines, respectively.

As it happened with the thermal denaturations followed by fluorescence, the transitions followed by the ellipticity at 222 nm did not show any sigmoidal behavior below pH 6.0 (Figure S7B), but above that pH there was an irreversible broad transition (Figure 2C, right axis, filled circles).

To sum up, the spectroscopic probes (intrinsic and ANS fluorescence, CD, and NMR) indicate that LrtA acquired a native, with well-folded regions (Figure S2) from pH 6.0 to 9.0.

### 2.3. LrtA Showed an Irreversible Complex Unfolding Equilibrium

As the thermal denaturations were irreversible (either followed by fluorescence or CD, Figure 2C and Figure S7), we tried to determine the conformational stability of LrtA by using GdmCl-denaturations followed by fluorescence and CD (urea-denaturations followed by fluorescence did not show any sigmoidal behavior, Figure S8A, inset). The tendency in both refolding and unfolding curves, followed by fluorescence and CD, was the same (Figure S8); however, the refolding CD results indicate that the final ellipticity, acquired by the native state, was not the same as that in the unfolding experiments. Moreover, the fluorescence refolding curves indicate that the value of the <1/λ> was different to that in the unfolding ones, even though we used the same protein concentration. Then, we conclude that there was a hysteresis behavior, and chemical denaturations were also irreversible, as it could be expected for a protein composed of several domains (see below, Section 2.4.).

In addition, comparison of the unfolding CD and fluorescence results suggest that the unfolding of LrtA was not a simple two-state process, as the denaturation curves by both techniques were different. Whereas fluorescence curves showed two transitions, CD reported a sole one, whose apparent midpoint did not overlap with that of the fluorescence (Figure S8). Fluorescence denaturation curves at several protein concentrations (in the range from 1.9 to 19 μM (in protomer units)) indicate that the first transition monitored corresponded to a protein-concentration-dependent process (Figure S8B inset), as at high protein concentrations (19 μM) two transitions were observed, with apparent midpoints around 1.0 and 2.0 M GdmCl. This result further confirms that LrtA was an oligomeric protein.

### 2.4. Sequence Properties and Molecular Modeling of LrtA

The primary structure of LrtA possesses a relatively large fraction of charged residues, both acidic and basic. Due to their high hydrophilicity, these residues tend to hamper the hydrophobic collapse and increase disorder in the protein backbone. Predictors of local disorder [26,27,28,29] based on a variety of physical properties (Figure S9) were used to estimate the propensity of the protein sequence to fold. There is a consensus indicating the region around residues 100–130 is highly disordered, together with a few other residues at both protein termini. Since the experimentally determined radius of monomeric LrtA has a value close to that of a compact protein (see Section 2.1., DOSY-NMR results), this may indicate that the region 100–130 was either a long, disordered loop within a single domain protein or a coil region separating two distinct but spatially close domains.

**Figure 3:**
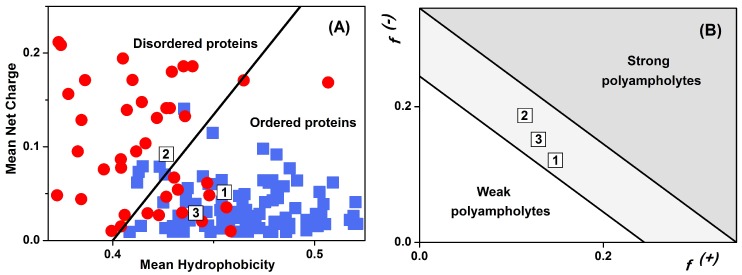
Location of LrtA in the diagram of state for charged polypeptides: Symbols “1”, “2”, and “3” indicate, respectively, the first two halves of the LrtA sequence (residues 1–100 and 101–191) and the whole protein. (**A**) Uversky plot based on the absolute mean net charge as a function of the mean scaled hydropathy, as obtained with PONDR [31]; well-folded (blue squares) and disordered proteins (red circles) are shown. (**B**) Das–Pappu plot based on the fraction f(+) and f(−) of positively and negatively charged residues, respectively [32].

The overall propensity of LrtA to fold into a well-structured protein was also explored by mapping its properties in terms of charge and hydropathy (Figure 3). In particular, Figure 3A compares the location of LrtA sequence within an Uversky diagram [30], which provides indication on the possibility that the protein belongs to the IDP (intrinsically disordered protein) class through the identification of a boundary hydropathy that separates folded and unfolded polypeptides. When the overall primary structure was considered, LrtA fell into the region of the diagram that is mostly populated by well-folded proteins [31], although also accessible to a few IDPs. However, when the sequence of LrtA was divided into two separate portions, they had distinct features. The first-half of LrtA sequence (residues 1–100) more distinctly belonged to the region occupied by ordered polypeptides. In contrast, the second-half (residues 100–191) fell in the region of the diagram that is populated by IDPs, although also accessible to some well-folded proteins. On the other hand, a Das–Pappu diagram [32] showed that LrtA should be considered a so-called ‘Janus sequence’ in between weak and strong polyampholytes (Figure 3B), independently whether the whole protein or just the two halves of its sequence are considered. This observation strongly suggests that the structure of LrtA is context-dependent, and may easily become more expanded/collapsed or structured/unstructured according to the environment (such as solution conditions or the presence of biomolecular partners). From our experimental results, LrtA had folded regions at pH 8.0 in aqueous solution (Figure S2 and Figure 2B, inset), although with a small stability, as suggested by thermal denaturations (Figure 2C and Figure S7A).

With the aim of building a model for the secondary and tertiary structures of LrtA, the protein sequence was submitted to full-chain protein structure prediction servers [33,34,35,36]. A particularly interesting result was obtained by using I-TASSER [33], which is one of the most popular and accurate software for generating high-quality model predictions of tridimensional protein structures. The best models predicted by I-TASSER (Figure S10) all included a well-structured domain spanning the first 100 residues, followed by a collapsed and poorly-structured region. The well-structured domain consisted of two parallel α-helices, and a β-sheet formed by four anti-parallel β-strands. These models were remarkable because they predicted a degree of order in the structure of LrtA that is in reasonable agreement with our expectations based on the CD experimental results (see above, Section 2.2.2), whereas in most cases the algorithms tend to overestimate the amount of secondary structure when applied to intrinsically unfolded polypeptides. Furthermore, the absence of a defined folding topology for the second half of LrtA sequence is consistent with the theoretical predictions discussed above. It is worth mentioning that the C-terminal region of HPF of *S. aureus*, another member of the long sub-family of HPF, in EM (electron microscopy) preparations was folded [5], in contrast with our model; then, it seems that in LrtA from *Synechocystis* sp. PCC 6803, the C-terminal region has specific features, which might be related to protein function. Finally, the conformations predicted for the first-half of LrtA sequence were in common with those obtained with the other algorithms of structural modeling that we used (i.e., FALCON [34], SWISS-MODEL [35], and Robetta [36]), although details of the geometry and orientation of the α-helices and β-strands were in some models different. This was particularly intriguing, especially because a four-strand motif is typical of many RNA-binding proteins ([5] and references therein).

Our theoretical predictions are not difficult to reconcile with the findings show by NMR, at physiological pH, where the spectrum of LrtA was that of a folded molecule (Figure S2). In fact, the signals of the proton nuclei in the unfolded and folded halves of the protein had a different behavior. The amide protons of the unfolded half of the protein would appear between 8.0 and 8.5 ppm [37], where they would be probably obscured, although they should be sharper than the rest of the signals, by many of the amide resonances of the folded half (those of the residues connecting the α-helices and the β-strands); it is interesting to note, however, the presence of a higher intensity at 8.2–8.3 ppm (Figure S2B), which could be due to the sharper resonance of the unfolded region of LrtA. Furthermore, the majority, if not all, of the amide protons of the unfolded half will be broadened and exchanged with the solvent at pH 8.0 [37], as it has been observed to occur in other intrinsically disordered regions, when the pH is raised and even when the temperature is decreased at the highest explored pH [38]. However, we tried to acquire a 1D ^1^H NMR spectrum at pH 6.9 (in the presence of 0.5 M NaCl) and 15 °C; under these conditions (Figure S11) some amide signals appearing between 7.8 and 8.3 became sharper, as expected for a disordered region that has a fast molecular tumbling. The methyl region, under these solvent conditions, was similar to that acquired at higher pH and temperature (Figure S2). In addition, all the methyl peaks corresponding to the side-chains of Val, Ile, and Leu residues of the disordered half of the protein under any of the conditions explored (pH 8.0, 20 °C or pH 6.9, 15 °C) would appear at basically the same chemical shifts as those of the corresponding folded region, i.e., around 0.8 ppm [37].

The models predicted by I-TASSER provided a static picture of LrtA that does not take into account its dynamics, which could be expected to be significant to determine the properties of such a chameleonic protein. Furthermore, the main difference between the predicted models and our experimental findings is the presence of a larger amount of β-structures in the former. Thus, we suspected that the structure predicted corresponded to the most stable structure that LrtA can assume, e.g., under ideal conditions in solution or when bound to a partner molecule, although, the experimental evidence (Figure 2C, Figures S7 and S8) suggests that this structure was not very stable. For those reasons, we used MD simulations to study the behavior of the protein structure both at room and high temperatures. The latter case corresponds to the simplest and most direct way to investigate the dynamics of a protein under non-native conditions [39], speeding up the sampling by overcoming the energetic barriers that restrain the structure in a given conformation.

**Figure 4:**
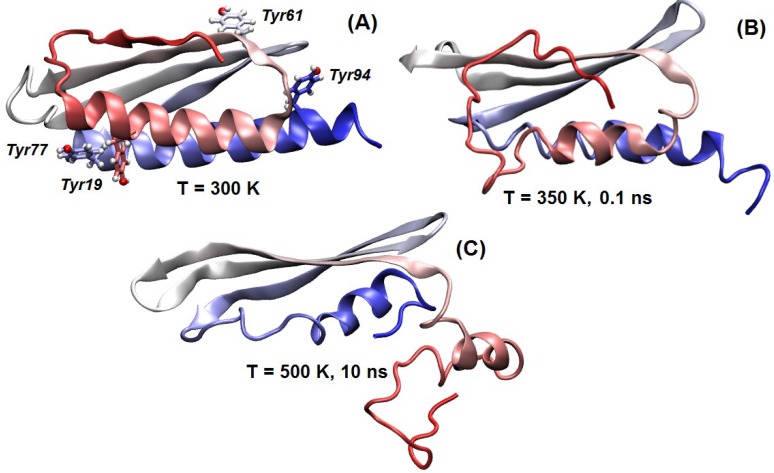
Dynamic behavior of the predicted folded domain of LrtA: The region comprising residues 1–100 of LrtA is shown in cartoon representation (colored from red (N terminus) to silver-white (mid-sequence regions) up to blue (C terminus of the domain)). (**A**) Structure at room temperature, with Tyr residues indicated. (**B**) Simulation under unfolding conditions: in the N-terminal region (indicated in red), the first β-strand loses it structure and coordination with the rest of the β-sheet. (**C**) Structure under extreme conditions: the folding topology is maintained. VMD [40] is used for the protein displays.

The MD results showed that the region including residues 1–100 is stable and maintains its folding topology and structure when simulated at room temperature (Figure S12). In particular, as shown in Figure 4, Tyr19 and Tyr77 interact to fix the two α-helices, whereas the other two Tyr residues are on the opposite face of the protein (Figure 4B). In contrast, when the temperature was raised, Tyr19 and Tyr77 lost their coordination and the first N-terminal β-strand immediately started losing its anchoring with the rest of the protein and the β-sheet scaffold (Figure 4B), increasing the amount of coil and helical structure. The anchoring was not recovered in annealing runs performed by reducing back the temperature, unless they were started at the earliest step of the local unfolding process. This finding suggests that the folding of the N-terminal region of LrtA was possibly assisted by interactions with other biomolecules, which may include other monomers of LrtA or binding partners, such as RNA. In contrast, the rest of the protein was very stable, and did not lose its folding topology even under the most extreme simulation conditions (Figure 4C).

## 3. Discussion

LrtA seemed to acquire a native-like conformation from pH 6.0 to 9.0. Changes in secondary (far-UV CD) and tertiary (intrinsic fluorescence) structures, and in the burial of hydrophobic residues (ANS fluorescence) occurred concomitantly at low pH. Under acidic conditions (pH 3.0), species with a higher amount of secondary structure (as indicated by a larger (in absolute value) ellipticity, Figure 2B) appeared to be populated, although they had hindered solvent-accessibility towards I^−^ and acrylamide quenchers (Table 1). Therefore, at low pH, LrtA had non-native conformations with non-stable secondary and tertiary structures (as judged by the absence of a sigmoidal shape in thermal denaturation curves). These results are further supported by the NMR spectra acquired at low pH, where there was no dispersion of amide or methyl signals (Figure S3), suggesting the presence of conformations with an unfolded structure. Besides, the broadening observed in the methyl and amide regions suggested the presence of aggregation, which was further confirmed by the far-UV CD spectra at pH 4.5 at different protein concentrations. Then, the protein at acidic pH had a larger tendency to associate than at physiological pH. The increase of ANS fluorescence at low pH, indicating a large solvent-accessible hydrophobic surface area, may appear difficult to reconcile with the results of I^-^ or acrylamide quenching, suggesting a smaller solvent accessibility towards Tyr (Table 1). However, the larger amount of hydrophobic surface area (monitored by ANS), that became solvent-exposed at acidic pH values, could involve several of the Val, Leu, and Ile residues, which are highly abundant in LrtA (46 out of 197 amino acids).

LrtA had well-folded regions in the pH range from 6.0 to 9.0, as indicated by: (i) the sigmoidal curves in the thermal and chemical denaturations (fluorescence and far-UV CD) (Figure 2C, Figure S7 and S8); and (ii) the 1D ^1^H-NMR spectrum at pH 7.0 and 8.0 (Figure S2). Moreover, the protein was capable of binding homogenous yeast RNA (from Sigma) with an affinity of ~1 μM (Figure S13), and therefore the purification protocol did not affect the conformational features of the protein. However, this structure was not highly rigid, as judged from the apparent thermal midpoint obtained from the irreversible denaturation curves (~40 °C, Figure 2C); these findings agree with the MD results in this work. As the secondary and tertiary structures of LrtA are only stable in a narrow thermal range, an increase in the temperature environment reduces the availability of well-folded and active protein, and therefore, a larger amount of protein is needed to carry out the cyanobacterial functions. The LrtA structure under native conditions, as suggested by the deconvolution of CD data, had a smaller percentage of α-helix structure than other HPF members (30% vs. 45%), as well as a lower percentage of β-sheet (17% vs. 27%). These experimental percentages were confirmed by the results of our MD simulations.

LrtA was an oligomeric protein at physiological pH. We showed that some of the Tyr residues seemed to be involved in the self-associating interface, as judged by the changes in the fluorescence lifetimes (Table 1) or the protein-concentration dependence of the curve denaturations midpoints (Figure S8B inset). In addition, our MD simulations at room temperature suggest that Tyr19 and Tyr77 in the folded domain of the protein were key in anchoring amino acids of the β-sheet, and the loss of such anchoring during the high-temperature simulations caused a partial disruption of the protein β-sheet. Then, Tyr residues were important for quaternary and secondary scaffolding in LrtA. Recently, it has been observed that, in EM preparations, the HPF of *S. aureus* (another member of the long HPF subfamily) is forming domain-swapped dimeric species [5]. Furthermore, crystals of the short HPF from *Vibrio cholerae* show the presence of dimers mediated by Co (II) anchoring residues of the β-sheets of two monomers [11], further pinpointing to the crucial importance of residues in the β-sheet for a possible quaternary arrangement of any HPF member. However, the importance of oligomerization for the function of all those proteins (included LrtA) remains to be elucidated, as it could be an adaptive mechanism of regulation to interact with other proteins or even with RNA.

## 4. Materials and Methods

### 4.1. Materials

Deuterium oxide and IPTG was obtained from Apollo Scientific (Stockport, UK). Sodium trimethylsilyl [2,2,3,3-^2^H_4_] propionate (TSP), imidazole, DNase, Trizma base and acid, yeast RNA, glutaraldehyde (25% *w*/*v* solution), ANS, deuterated acetic acid, its sodium salt and His-Select HF nickel resin were from Sigma-Aldrich (Madrid, Spain). The β-mercaptoethanol (β-ME) was from BioRad (Madrid, Spain). Triton X-100 and protein marker, PAGEmark-tricolor (G Biosciences) were from VWR (Barcelona, Spain). Dialysis tubing, with a molecular weight cut-off of 3500 Da, was from Spectrapor (Spectrum Laboratories, Breda, The Netherlands). Amicon centrifugal devices with a cut-off molecular weight of 3000 Da were from Millipore (Barcelona, Spain). Standard suppliers were used for all other chemicals. Water was deionized and purified on a Millipore system.

### 4.2. Protein Expression and Purification

Expression of LrtA was carried out in BL21(DE3) or C41 [41] strains with a final ampicillin concentration of 100 mg/mL at 37 °C. The cells were cultured in 1 L flasks. Protein expression was induced with a final concentration of 1.0 mM IPTG when the absorbance of the cell culture at 600 nm was 0.4–0.9, and the cells were grown for 15–16 h at 37 °C. Cells were harvested at 8000 rpm in a JA-10 rotor (Beckman Coulter, Miami, FL, USA) for 15 min. The pellet from 5 L of culture was re-suspended in 50 mL of buffer A (500 mM NaCl, 5 mM imidazole, 20 mM Tris buffer (pH 8), 0.1% Triton X-100 and 1 mM β-ME), supplemented with a tablet of Sigma Protease Cocktail EDTA-free and 2 mg of DNase (per 5 L of culture). After being incubated with gentle agitation at 4 °C for 10 min, cells were disrupted by sonication (Branson sonicator, 750 W, Richmond, VA, USA), with 10 cycles of 45 s at 55% of maximal power output and an interval of 15 s between the cycles. All the sonication steps and the interval waits were carried out in ice. The lysate was clarified by centrifugation at 18,000 rpm for 40 min at 4 °C in a Beckman JSI30 centrifuge with a JA-20 rotor (Beckman Coulter, Miami, FL, USA).

The clarified lysate from such first centrifugation did not contain a large amount of LrtA, and thus, we suspected that most of the protein was present in the cell debris precipitate. Therefore, the precipitate was treated with buffer A supplemented with 8 M urea and a tablet of Sigma Protease Cocktail EDTA-free and 2 mg of DNase (per 5 L of culture). The re-suspended sample was treated with another 10 cycles of sonication in ice, and the sample was clarified by centrifugation at 20,000 rpm for 30 min at 4 °C. LrtA was in the supernatant and was purified by immobilized affinity chromatography (IMAC). The supernatant was added to 5 mL of Ni-resin previously equilibrated in buffer A supplemented with 8 M urea. The mixture was incubated for 20 min at 4 °C, and afterwards, the lysate was separated from the resin by gravity. On-column refolding was carried out during the washing step with 20 mL of buffer B (20 mM Tris buffer (pH 8.0), 500 mM NaCl, 1 mM β-ME, and 20 mM imidazole); the protein was eluted by gravity from the column with buffer C (20 mM Tris buffer (pH 8.0), 500 mM NaCl, 1 mM β-ME, and 500 mM imidazole). The eluted LrtA was extensively dialyzed against buffer D (100 mM sodium phosphate buffer (pH 8.0) with 500 mM NaCl). Precipitate in the dialysis tubing after five dialysis steps in buffer D was removed by centrifugation at 20,000 rpm for 30 min at 4 °C. The final yield of protein was 4.5–6.5 mg/L of culture (with both cellular strains assayed), and the protein was 90–95% pure as judged by SDS gels (Figure S14). This purity percentage takes into account the possible contamination due to the presence of deoxyribonucleotides, as judged by their absorbance at 260 nm (see below).

We attempted to re-purify the protein recovered from IMAC by using gel filtration chromatography in a Superdex 16/600, 75 pg column (GE Healthcare, Barcelona, Spain) connected to an AKTA FPLC system (GE Healthcare) by monitoring the absorbance at 280 nm; nevertheless, the protein was bound to the column and did not come out within its bed volume. Binding to the column has been also observed during purification of the recombinant HPF from *Staphylococcus aureus* [5], another member of the long subfamily of HPF.

The eluted protein from IMAC showed absorbance at 260 nm, suggesting that it was probably contaminated with di- or tri-deoxyribonucleotides (from the cleavage of the DNase used during purification and even though sample was dialyzed against 500 mM of NaCl). Presence of deoxyribonucleotides has been also observed in the recombinant HPF from *S. aureus* after its purification [5]. We tried to remove the deoxyribonucleotides by using different concentrations of polyethylenimine (PEI), ranging from 0.2 to 1% (*v*/*v*) [42], but most of LrtA co-precipitated with the oligonucleotides. The total protein concentration, *P*_c_ (in mg/mL) was determined by using the expression [43]: *P*_c_ = 1.55 A_280_ − 0.75 A_260_, where A_280_ and A_260_ are the absorbance of the dialyzed protein solution at 280 and 260 nm, respectively. However, it is important to note that the presence of deoxyribonucleotides did not affect the spectroscopic signals either of both fluorescence and far-UV CD, because DNA is spectroscopically silent in fluorescence, and in the far-UV region of CD spectra between 210–240 nm, deoxyribonucleotides do not absorb [44,45]. It is also interesting to note that deoxyoligonucleotides in aqueous solution or intact DNA show a small or slightly positive ellipticity around 222 nm [45,46,47,48,49] where we have carried out the study of the CD biophysical properties of the protein (see Results section).

### 4.3. Fluorescence

Fluorescence spectra were collected on a Cary Varian spectrofluorimeter (Agilent, Foster City, CA, USA), interfaced with a Peltier, at 25 °C. LrtA concentration in the pH- or chemical-denaturation experiments was 9.8 μM (in protomer units). For experiments with ANS, a final probe concentration of 100 μM was added. A 1-cm-pathlength quartz cell (Hellma, Mullheim, Germany) was used.

In the pH-induced unfolding curves, the pH was measured after completion of the experiments with an ultra-thin Aldrich electrode in a Radiometer pH-meter (Madrid, Spain). The acids and salts used were: pH 2.0–3.0, phosphoric acid; pH 3.0–4.0, formic acid; pH 4.0–5.5, acetic acid; pH 6.0–7.0, NaH_2_PO_4_; pH 7.5–9.0, Tris acid; pH 9.5–11.0, Na_2_CO_3_; pH 11.5–13.0, Na_3_PO_4_. Chemical and pH denaturations were repeated three times with new samples at any of the concentrations assayed. Appropriate blank corrections were made in all spectra both in pH- and chemical- denaturation experiments.

For GdmCl-denaturation experiments the samples were prepared the day before from a 7 M GdmCl concentrated stock and left overnight to equilibrate; before experiments, samples were left to 25 °C for 1 h. For the refolding experiments, the sample was exchanged in 7 M GdmCl by using Amicon centrifugal devices; protein concentration was the same as in the unfolding experiments.

The emission intensity weighted average of the inverse wavelengths (also called the spectrum mass center, or the spectral average energy of emission), <1/λ>, was calculated as described [50]. Briefly, we define <1/λ> as: $\langle 1/\lambda \rangle =\sum_{1}^{n}\frac{1}{\lambda_{i}}I_{i}/\sum_{1}^{n}I_{i}$, where *I*_i_ is the intensity at wavelength λ_i_. We shall report <1/λ> in units of μm^−1^.

Steady-State Spectra—The experimental set-up for the intrinsic and ANS fluorescence pH-denaturation experiments has been described previously [50]. Briefly, protein samples were excited at 278 nm, for the intrinsic fluorescence, and 380 nm for the ANS experiments. In all cases, excitation and emission slits were 5 nm. The experiments were recorded between 300 and 400 nm (for the intrinsic fluorescence) and between 400 to 600 for the ANS experiments. The signal in all cases was acquired for 1 s and the increment of wavelength was set to 1 nm. For the chemical denaturations, following intrinsic fluorescence, several protein concentrations were used in the range from 1.9 to 19.6 μM (in protomer units) at 100 mM phosphate buffer (pH 7.0) and 50 mM NaCl.

Thermal Denaturations—Thermal denaturations of isolated LrtA at different pH values were carried out with the same experimental set-up described [50] and protein concentrations of 9.8 and 5 μM (in protomer units). Briefly, these experiments were performed at constant heating rates of 60 °C/h and an average time of 1 s. Thermal scans were collected at 308 nm after excitation at 278 nm from 25 to 95 °C and acquired every 0.2 ° C.

Fluorescence Quenching—Quenching by iodide and acrylamide was examined at different solution conditions, with an LrtA concentration of 9.8 μM (in protomer units): pH 3.5 (formic buffer, 50 mM), pH 7.0 (phosphate buffer, 50 mM), and pH 11.0 (boric buffer, 50 mM). Experiments were also carried out in the presence of 6 M GdmCl at pH 7.0 (50 mM, phosphate buffer). The experimental set-up for both quenchers was the same described above for the intrinsic fluorescence experiments. The data for KI were fitted to [12]

(1) F0F=1+Ksv[KI]

where *K*_sv_ is the Stern-Volmer constant for collisional quenching; *F_0_* is the fluorescence intensity in the absence of KI; and *F* is that at any KI concentration. The range of KI concentrations explored was 0–0.7 M. For experiments with acrylamide, the data were fitted to [12]

(2) F0F=(1+Ksv[acrylamide])eυ[acrylamide]

where υ is the dynamic quenching constant. Fittings to Equations (1) and (2) were carried out by using Kaleidagraph (Synergy software, Dubai, United Arab Emirates).

### 4.4. Fluorescence Lifetimes

Lifetimes were measured on an EasyLife V™ lifetime fluorometer (Madrid, Spain) with the stroboscopic technique, by using as excitation source a pulsed light of a diode LED operating at 278 nm. The number of channels used for each scan was 500, and the integration time was 1 s. Three scans were averaged in each experiment, and they were repeated twice at 25 °C in 50 mM phosphate buffer (pH 8.0) and 500 mM NaCl.

The experimental fluorescence decays (*D*(*t*)) were fitted to a sum of exponential functions: $D(t)=\sum_{i=1}^{n}a_{i}\exp\left(-t/\tau_{i}\right)$, where *τ*_i_ is the the lifetime of the electronic excited states of the fluorescent species present in solution, and *a_i_* the pre-exponential factor of those electronic states. The pre-exponential factors can be interpreted not only in terms of the populations of the corresponding species, but also in terms of the radiative probability constants of Tyr residues, in the case of LrtA. We also determined the mean lifetime, <*τ*>, as: $<\tau {>}^{}{=}^{}\sum_{i=1}^{n}f_{i}\tau_{i}$, where the *f_i_*s are defined as: $f_{i}=a_{i}\tau_{i}/\sum_{j}a_{j}\tau_{j}$. The fitting procedure of the experimental fluorescence lifetime curves used an iterative method based on the Levenberg–Marquardt algorithm [51]. The temporal width of the excitation pulse, which distorted the observed decay, was taken into account through the instrument response function (IRF), which was determined by using a scattered solution of Ludox. Goodness of the fittings was tested by using a reduced χ^2^, that was calculated by measuring the spectral noise at time *t*, and determining the measurement uncertainties [52].

### 4.5. CD

The far-UV CD spectra were collected on a Jasco J815 spectropolarimeter (Jasco, Tokyo, Japan) fitted with a thermostated cell holder, and interfaced with a Peltier unit, at 25 °C. The instrument was periodically calibrated with (+)-10-camphorsulphonic acid. Several protein concentrations of LrtA (4.5, 9.8 and 19.6 μM, in protomer units) were used to test for concentration-dependent changes in the shape and intensity of the steady-state spectrum at two different pH values; differences were not observed at these concentrations (9.8 and 19.6 μM) at pH 8.0. However, protein-concentration-dependent changes (in the concentration range of 4.5 and 9.8 μM, in protomer units) were observed in the shape and ellipticity at pH 4.5, suggesting that at this pH the protein showed a larger tendency to self-associate (see above, Section 2.1, Figure S4) than at pH 8.0. Molar ellipticity was calculated as described [44,45,50].

Steady-State Spectra—Experiments were acquired with the same experimental set-up described previously [50]. Typically, spectra were acquired at a scan speed of 50 nm/min with a response time of 2 s and averaged over six scans with a bandwidth of 1 nm. Spectra were corrected by subtracting the baseline in all cases. Protein concentrations were 9.8 μM (in protomer units) for pH- and chemical-denaturation experiments (for both unfolding and refolding). The refolding samples of LrtA were prepared as described above for fluorescence.

Thermal Denaturations—Thermal denaturations were carried out with the same experimental set-up described previously [50] and a protein concentration of 9.8 μM. Briefly, thermal denaturations were performed at a constant heating rate of 60 °C/h, a response time of 8 s, a band width of 1 nm, and acquired every 0.2 °C. Thermal scans were collected in the far-UV region at 222 nm from 25 to 85 °C.

### 4.6. NMR Spectroscopy

The NMR experiments were acquired at 20 °C or 15 °C, when stated, on a Bruker Avance DRX-500 spectrometer equipped with a triple resonance probe and z-pulse field gradients.

1D ^1^H-NMR experiments—Homonuclear 1D-^1^H-NMR experiments were performed with LrtA at a concentration of 30 μM (in protomer units) in 0.5 mL, in 100 mM phosphate buffer (pH 8.0 or pH 6.9) and 500 mM NaCl in H_2_O/D_2_O (90%/10%, *v*/*v*), uncorrected for deuterium isotope effects. The 1D-^1^H spectra under these conditions were acquired with 16 K data points, with 2 K scans and a 6000 Hz spectral width (12 ppm), with the WATERGATE sequence [53]. Baseline correction and zero-filling were applied before processing. We also acquired a spectrum at pH 4.5 (100 mM acetate) with 64 K scans, while the other experimental parameters were as above. During buffer exchange in Amicon centrifugal devices the sample precipitated and we had to increase the number of scans to have a good signal-to-noise ratio. All spectra were processed and analyzed by using TopSpin 2.1 (Bruker GmbH, Karlsruhe, Germany). TSP was used as the external chemical shift reference, taking into account the pH-dependence of its resonance [15].

Translational Diffusion Measurements*—*The DOSY experiments at pH 8.0 and 4.5 were performed with the pulsed-gradient spin-echo sequence, as described previously [50], with 16 gradient strengths ranging linearly from 2 to 95% of the total power of the gradient unit. At both pH values, the duration of the pulse gradient was 2.5 ms, and the time between the two gradients was 150 ms. Samples were exchanged in D_2_O buffer (100 mM phosphate buffer (pH 8.0, not corrected by isotope effects) or 100 mM deuterated acetate buffer (pH 4.5, not corrected by isotope effects) with 500 mM NaCl at both pH values) by using Amicon centrifugal devices during 4 to 6 h. Precipitation was observed after the buffer exchange (from pH 8.0 to pH 4.5), and the sample was centrifuged at 13,000 rpm for five minutes, before putting it into the NMR tube. Further and more severe precipitation was observed during the D_2_O buffer exchange at pH 4.5 and attempts to acquire a DOSY by increasing the number of scans (until 8 K) at each pulse field gradient strength failed. The methyl region was used for intensity measurements in the experiment acquired at pH 8.0.

### 4.7. DLS

DLS measurements were performed at 25 °C in 100 mM phosphate buffer (pH 8.0) with 500 mM NaCl at different protein concentrations. Experiments were performed at fixed angle (θ = 173°) in a Zetasizer nano instrument (Malvern Instrument Ltd., Malvern, UK) equipped with a 10-mW helium-neon laser (λ = 632.8 nm) and a thermoelectric temperature controller. Experiments were analyzed with Zetasizer software (Malvern Instrument Ltd., Malvern, UK), and they were carried out as described [50]. Each sample was measured 10 times with 10 runs of 30 s each. The Z-average size was obtained by fitting the autocorrelation function with the cumulants method. The *R*_S_ was calculated by applying the Stokes–Einstein equation: *D = kT/*6πη*R*_S_, where *k* is the Boltzmann constant and *T* is the temperature (in K). Experiments were also carried out at pH 4.5 (100 mM acetic acid) and 500 mM NaCl, but the poor signal obtained precluded any reliable conclusion.

### 4.8. Molecular Modelling

Disorder propensity was estimated by using scoring functions according to independent algorithms [26,27,28,29], as calculated through submission to their respective web servers. Three-dimensional models of the protein were obtained using the on-line structure predictors I-TASSER [33], FALCON [34], SWISS-MODEL [35], and Robetta [36]. Folding stability in the LrtA structure predicted by I-TASSER was assessed in MD simulations at room temperature performed with the GROMACS package [54]. The AMBER ff99SB-ILDN force field [55] and TIP3P model [56] were used for the protein and water, respectively, and other simulation conditions were as previously described [57,58]. High temperature simulations were performed at 400 and 500 K, according to a protocol previously adopted [59].

### 4.9. SEC

SEC experiments were carried out in a Superose 12 10/300 GL column at pH 8.0 (50 mM Tris) and 0.7 M NaCl, connected to an AKTA FPLC (GE Healthcare), and monitoring the absorbance at 280 nm. Flow rate was 0.7 mL/min, and the protein was loaded in a volume of 100 μL at a concentration of 97 μM (in protomer units). Baselines of the chromatograms were strongly curved and therefore the UNICORN software 5.0.2 (GE Healthcare, Barcelona, Spain) was used for automatic baseline correction. The markers used to calibrate the column were albumin, bovine RNase A and blue dextran (GE Healthcare); the isolated markers were loaded in the column in the above described buffer in three independent measurements.

### 4.10. Glutaraldehyde Cross-Linking

Glutaraldehyde cross-linking was carried out at pH 8.0 (50 mM Tris buffer) in the presence of 0.5 M NaCl at room temperature. Sample volume was 200 μL. Protein concentration was 97 μM (in protomer units). The protocol used was that described previously [60], taking aliquots of a fresh 25% (*w*/*v*) glutaraldehyde solution (Sigma-Aldrich) to yield a final concentration of the cross-linker in the protein solution of 1%. Aliquots of 30 μL were extracted at defined times and the cross-linking reaction was stopped by adding an identical volume of SDS-loading buffer to the aliquot. The extracted samples were run in a 12% SDS-PAGE gel immediately. Experiments were repeated twice.

## 5. Conclusions

Our results show that LrtA from cyanobacteria, a member of the long HPF subfamily, shows a clear propensity to self-associate and has an apparent low conformational stability. The structure was predicted to be formed by two domains, one of which was well-folded, whereas the other was disordered. These conformational features, together with the presence of a relatively high number of charged residues, provide an overall structural plasticity.

## Figures and Tables

**Table 1:** Fluorescence lifetimes of LrtA (100 mM, phosphate buffer (pH 8.0), with 500 mM NaCl) at 25 °C ^a^.

Concentration (μM)	*τ*_1_ (ns)	a_1_	*τ*_2_ (ns)	a_2_	<*τ*> (ns)	χ^2^
98	0.49 ± 0.08	1.9 ± 0.2	3.4 ± 0.1	0.072 ± 0.005	1.101	1.384
9.8	0.46 ± 0.07	2.0 ± 0.2	3.6 ± 0.2	0.048 ± 0.006	0.9622	1.528
7.8	0.54 ± 0.08	1.8 ± 0.2	2.7 ± 0. 2	0.07 ± 0.01	0.9037	1.35
1.9	0.69 ± 0.09	0.71 ± 0.09	14.9 ± 0. 8	0.0156 ± 0.0008	5.233	1.22
0.98	0.48 ± 0.07	1.0 ± 0.1	19.5 ± 0.4	0.0104 ± 0.0005	5.996	1.238

^a^ Errors are from fitting to a bi-exponential function.

**Table 2:** Quenching parameters for LrtA under several conditions at 25 °C.

Solution Conditions	KI	Acrylamide	
	*K*_sv_ (M^−1^)	*K*_sv_ (M^−1^)	υ (M^−1^)
pH 3.0	- ^a^	7.7 ± 0.5	2.1 ± 0.1
pH 7.0	1.63 ± 0.03	11 ± 1	2.2 ± 0.3
pH 11	0.78 ± 0.02	9.7 ± 0.5	0.5 ± 0.1
GdmCl (pH 7.0)	3.1 ± 0.1	22 ± 4	2.0 ± 0.3

^a^ Not determined due to protein precipitation.

## Supplementary Materials

The following are available online at http://www.mdpi.com/1422-0067/19/7/1857/s1, There are 14 figures, showing: the intensity curve of the methyl groups from the DOSY experiment at pH 8.0 and 500 mM NaCl (Figure S1); the spectroscopic characterization of LrtA by NMR at pH 8.0 and 500 mM NaCl and 20 °C (Figure S2); the 1D 1H-NMR spectrum of LrtA at pH 4.5 and 500 mM NaCl and 20 °C (Figure S3); far-UV CD spectra of LrtA at pH 4.5 at two different concentrations (Figure S4); the SDS-PAGE gel with the glutaraldehyde cross-linking results (Figure S5); the fluorescence lifetime at pH 8.0 and 500 mM NaCl of LrtA at 0.98 μM (in protomer units) (Figure S6); the thermal denaturations followed by fluorescence at different pH values (Figure S7); the chemical (urea and GdmCl) denaturations of LrtA followed by fluorescence and CD (Figure S8); the predictions of disorder probability along the sequence (Figure S9); several models of the LrtA structure as obtained from homology modeling (Figure S10); the spectroscopic characterization of LrtA by NMR at pH 6.9 and 500 mM NaCl at 15 °C (Figure S11); the secondary structure of the protein obtained in MD simulations (Figure S12); the titration of LrtA to yeast RNA (Figure S13); and the SDS-gel of purified LrtA (Figure S14).

## Abbreviations

ANS	8-anilino-1-naphthalene sulfonic acid
β-ME	β-mercaptoethanol
CD	circular dichroism
DLS	dynamic light scattering
DOSY	diffusion ordered spectroscopy
EM	electron microscopy
GdmCl	guanidine hydrochloride
HPF	hibernating promoting factor
IDP	intrinsically disordered protein
IMAC	immobilized affinity chromatography
IRF	instrument response function
ITC	isothermal titration calorimetry
MD	molecular dynamics
NMR	nuclear magnetic resonance
PEI	polyethylenimine
RMF	ribosome modulation factor
RNase	ribonuclease
SDS-PAGE	sodium dodecyl sulfate polyacrylamide gel electrophoresis
SEC	size exclusion chromatography
UV	ultraviolet
