# Supplementary material for: The Cyanobacterial Ribosomal-Associated Protein LrtA from Synechocystis sp. PCC 6803 Is an Oligomeric Protein in Solution with Chameleonic Sequence Properties
Source: Int J Mol Sci. 2018 Jun 24;19(7):1857. doi: 10.3390/ijms19071857 (PMC6073757; doi:10.3390/ijms19071857)

International Journal of *Molecular Sciences*

MDPI

The cyanobacterial ribosomal-associated protein LrtA from *Synechocystis* sp PCC 6803 is an oligomeric protein in solution with chameleonic sequence properties

Lellys M. Contreras, Paz Sevilla, Ana Cámara-Artigas, José G. Hernández-Cifre, Bruno Rizzuti, Francisco J. Florencio, M. Isabel Muro-Pastor, José García de la Torre, and José L. Neira

SUPPLEMENTARY FIGURE LEGENDS

FIGURE S1: **DOSY experiments.** Intensity decay (y-axis, arbitrary units) of the methyl signals of the spectrum shown in Fig. S3 A, as the pulse field gradient strength was increased (x-axis). The line is the fitting to $\frac{I}{I_0} = -exp\left(D\gamma_H^2\delta^2G^2\left(\Delta - \frac{\delta}{3} - \frac{\tau}{2}\right)\right)$, where *I* is the peak intensity of a particular (or a group of) resonance(s); I_0 is the maximum peak intensity of the same resonance(s) at the smallest gradient strength; δ is the duration (in s) of the gradient; *G* is the gradient strength (in T cm^{-1}); Δ is the time (in s) between the gradients; γ_H is the gyromagnetic constant of the proton; and, τ is the recovery delay between the bipolar gradients (100 μs). The fitting to the experimental intensities was obtained with Kaleidagraph (Synergy software). DOSY experiments were acquired in 100 mM phosphate buffer (pH 8.0), with 500 mM NaCl at 20 ºC.

FIGURE S2: **1D ^{1}H-NMR characterization of LrtA at pH 8.0.** Methyl (A) and amide (B) proton regions of the ^{1}H-NMR spectrum of LrtA. The intensity of panel (B) has been increased twenty-times compared to that of panel (A). Spectrum was acquired in 100 mM phosphate buffer with 500 mM NaCl at 20 ºC.

FIGURE S3: **1D ^{1}H-NMR characterization of LrtA at pH 4.5.** Methyl (A) and amide (B) proton regions of the ^{1}H-NMR spectrum of LrtA. The large peaks appearing around 2.0 ppm and 1.2 ppm correspond, respectively, to the residual non-deuterated acetic acid (from the buffer used) and to "fingertip" contamination (probably lactic acid or alanine). The enlargement of the intensity used in panel (B) was forty times that of panel (A). Spectrum was acquired in 100 mM acetate buffer with 500 mM NaCl at 20 ºC.

FIGURE S4: **Far-UV CD spectra at pH 4.5 and 500 mM NaCl.** Far-UV CD spectra at two different LrtA concentrations at 25 ºC.

FIGURE S5: **Self-association of LrtA monitored by cross-linking.** 12 % SDS-PAGE of the products of glutaraldehyde cross-linking at different reaction times. Final concentration of glutaraldehyde was 1 %. The marker used was PAGEmark-Tricolor (in lane (1)), with markers (from top to bottom) of 210, 110, 67, 48, 32, 10, 16 and 6 kDa. The lanes shows the results of the cross-linking reaction after 5 min (lanes (2) and (7)); 30 minutes (lane (3); 1 hour (lane (4) and (6)); and 90 minutes (lane (5)).

FIGURE S6: **Fluorescence lifetime curves of LrtA.** Fluorescence lifetime decay at pH 8.0 with 0.5 M NaCl at 25 ºC, at a protein concentration of 0.98 μM (in protomer units). The red curve is the fitting to a bi-exponential function.

FIGURE S7: **Thermal denaturations of LrtA at several pH values.** The thermal unfolding of LrtA at several pHs as monitored by fluorescence emission at 308 nm (A), and by ellipticity at 222 nm (B) in the presence of 0.5 M NaCl at 9.8 μM.

FIGURE S8: **Chemical denaturation of LrtA.** (A) Unfolding of LrtA followed by the changes in <1/λ> (blue circles) and refolding (black, blank circles) at pH 7.0 (50 mM, phosphate buffer) with 50 mM NaCl and 25 ºC at 9.8 μM (protomer units). Inset: Urea-denaturations of LrtA followed by the changes in intensity at 308 nm at pH 7.0 (50 mM, phosphate buffer) with 50 mM NaCl and 25 ºC at 9.8 μM (protomer units). (B) Unfolding of LrtA followed by the changes in the ellipticity at 222 nm (blue circles) and refolding (black circles) at pH 7.0 (50 mM, phosphate buffer) with 50 mM NaCl and 25 ºC and 9.8 μM (protomer units). The units of ellipticity are in mdeg as provided by the instrument. Inset: GdmCl-denaturations followed by fluorescence at different LrtA concentrations (in protomer units indicated in the figure).

FIGURE S9: **Predicted disordered regions in LrtA sequence.** Amino acids are classified as prone to either disorder (score > 50%) or order (score < 50%) by using the web-server predictors RONN [27], PrDOS [28], IUPred [26] and IsUnstruct [29].

FIGURE S10: **Predicted models of LrtA structure.** Best five models predicted by I-TASSER [32], all showing a well-structured domain including two parallel α-helices and a β-sheet formed by four anti-parallel β-strands, followed by a collapsed unstructured domain.

FIGURE S11: **1D ^{1}H-NMR characterization of LrtA at pH 6.9.** Methyl (A) and amide (B) proton regions of the ^{1}H-NMR spectrum of LrtA. The intensity of panel (B) has been increased twenty-five-times compared to that of panel (A). Spectrum was acquired in 100 mM phosphate buffer with 500 mM NaCl at 15 ºC.

FIGURE S12: **Structure of LrtA.** Structure of LrtA (in different colours) during the MD simulations at room temperature.

FIGURE S13: **Binding of LrtA to RNA.** Changes in the fluorescence intensity of LrtA upon yeast RNA addition. Experiments were carried out at pH 7.0 (50 mM, phosphate buffer) with 50 mM NaCl and 25 ºC. Increasing amounts of RNA (from Sigma-Aldrich), in the range 0-4 μM, were added to a solution with a fixed concentration of LrtA (3.5 μM, protomer units). The curve is the fitting to:

$$F = F_0 + \frac{\Delta F_{\max}}{2[LrtA]_T}\left[\left([LrtA]_T + [\mathrm{RNA}]_T + K_d\right) - \left(\left([LrtA]_T + [\mathrm{RNA}]_T + K_d\right)^2 - 4[LrtA]_T[\mathrm{RNA}]_T\right)^{1/2}\right],$$

where F is the measured fluorescence at any given concentration of RNA after subtraction of the blank; ΔF_{max} is the maximal change in the fluorescence of LrtA when all the RNA was forming the complex compared to the fluorescence of isolated LrtA; F_0 is the fluorescence intensity when no RNA was added; $[\mathrm{LrtA}]_T$ is the constant, total concentration of LrtA; and $[\mathrm{RNA}]_T$ is that of yeast RNA, which was varied during the titration.

FIGURE S14: **SDS-PAGE gel of LrtA purification.** The marker used was PAGEmark-Tricolor (in lanes (3) and (6)), with markers (from top to bottom) of 210, 110, 67, 48, 32, 10, 16 and 6 kDa. The theoretical molecular weight of LrtA was 22.7 kDa, and the purified protein is shown in lanes (2) and

(5). The C-terminal region of LrtA (residues-103-191), with a molecular weight of 12.5 kDa, is shown in lanes (1) and (4). Bis-acrylamide concentration of the gel was 12%.

Fig. S1 (Contreras et al.)

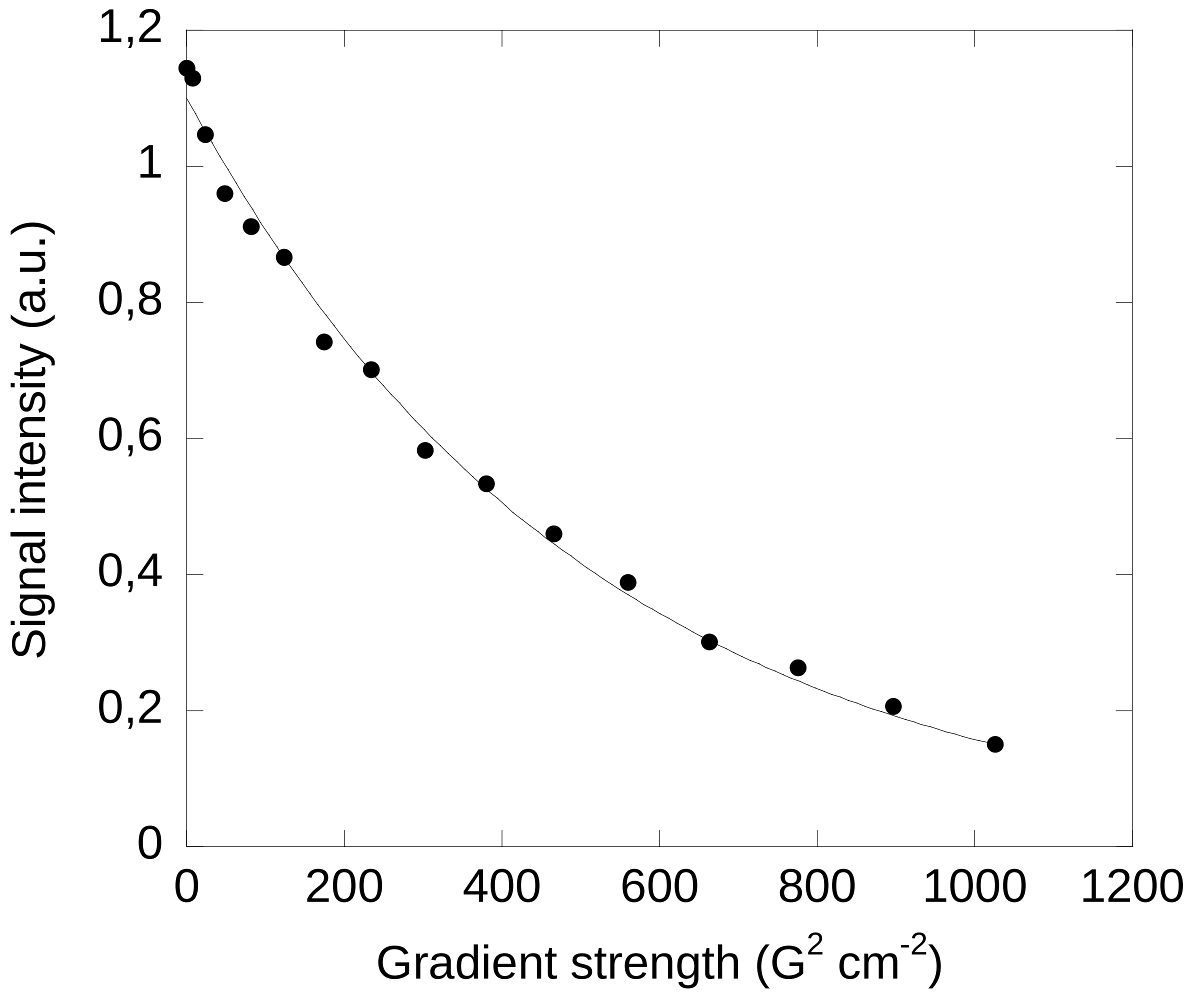

Fig. S2 (Contreras et al.)

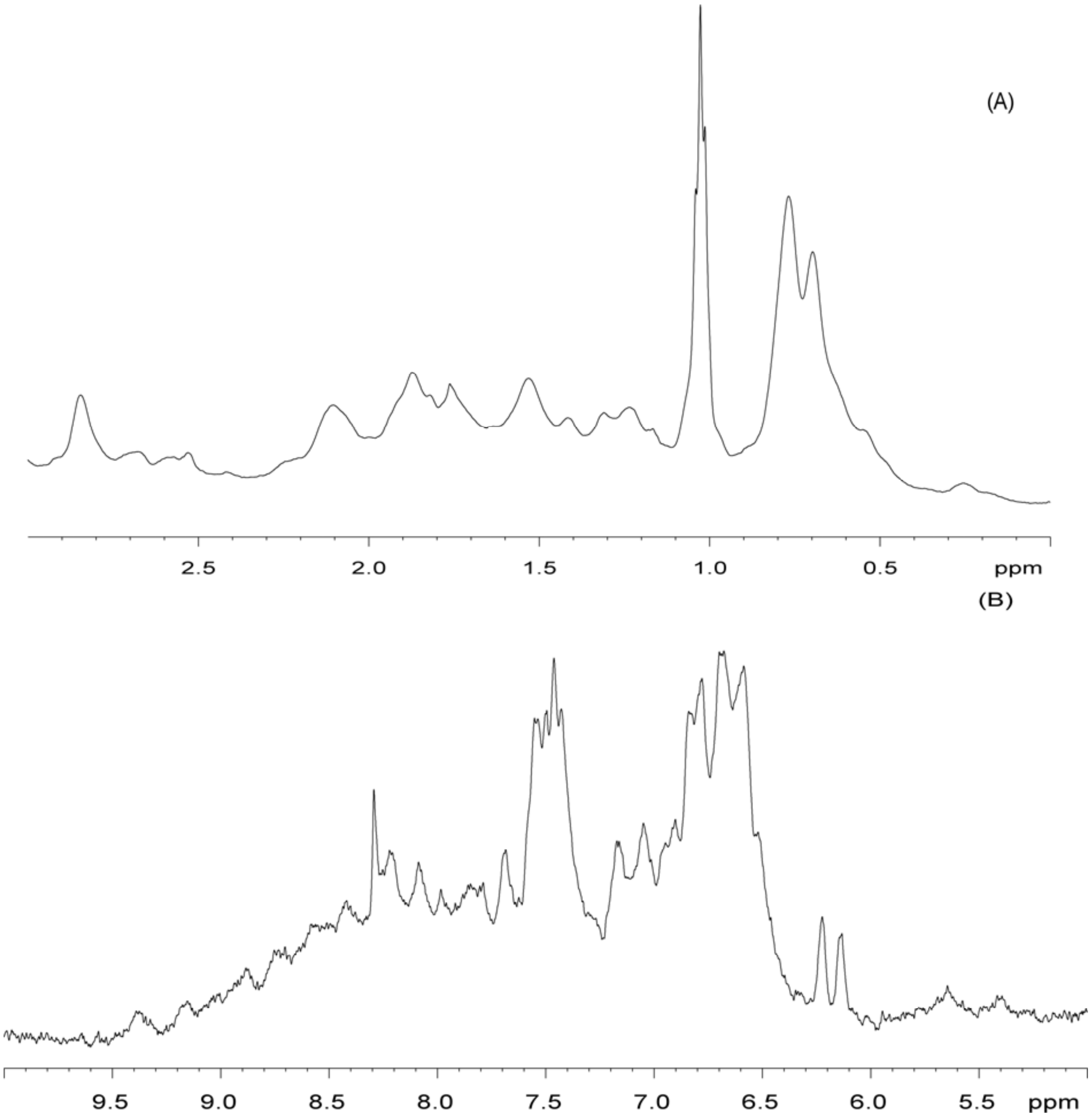

Fig. S3 (Contreras et al.)

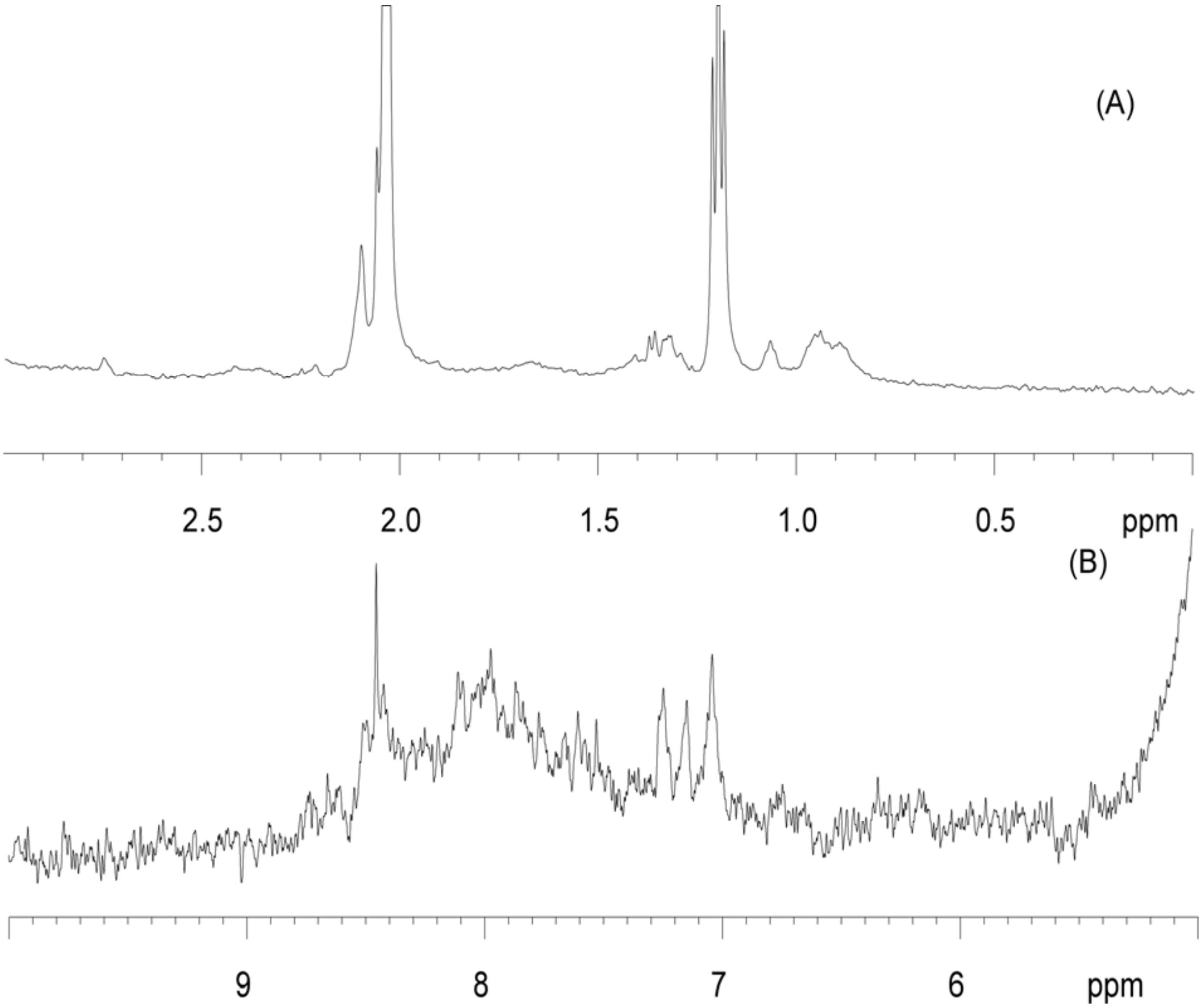

Fig. S4 (Contreras et al.)

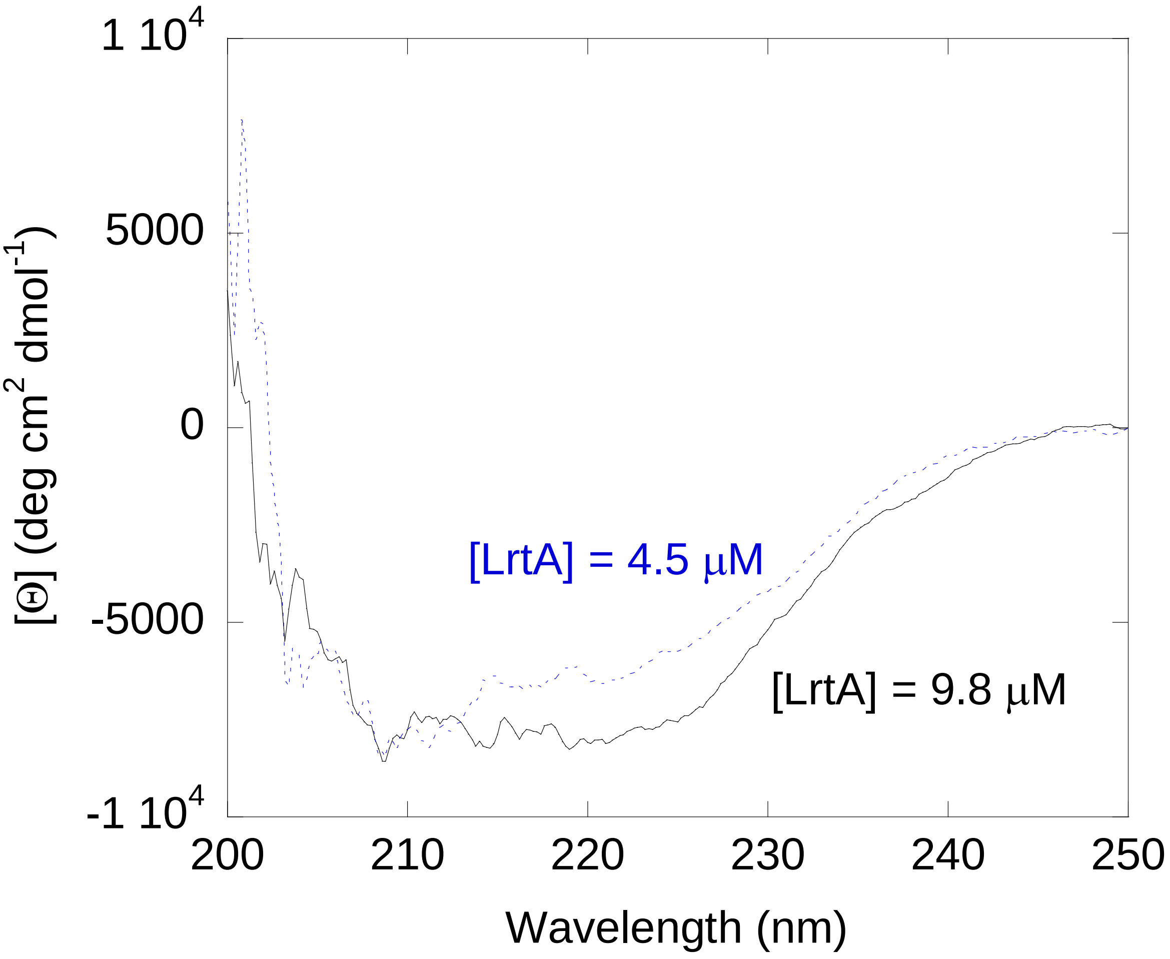

Fig. S5 (Contreras et al.)

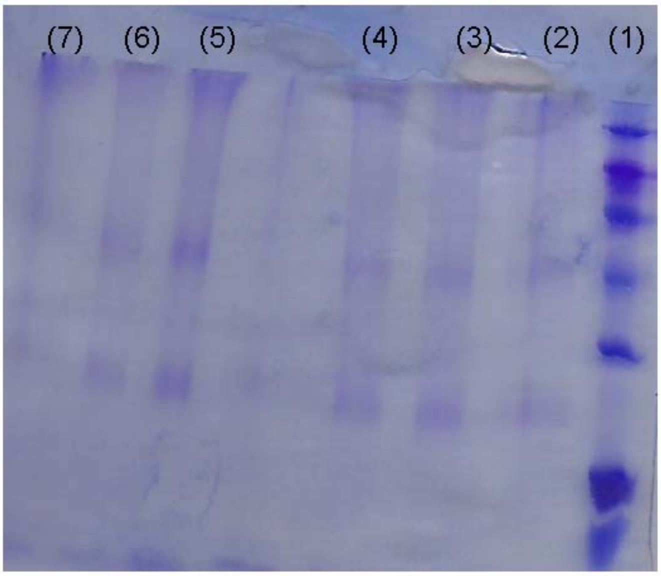

Fig. S6 (Contreras et al.)

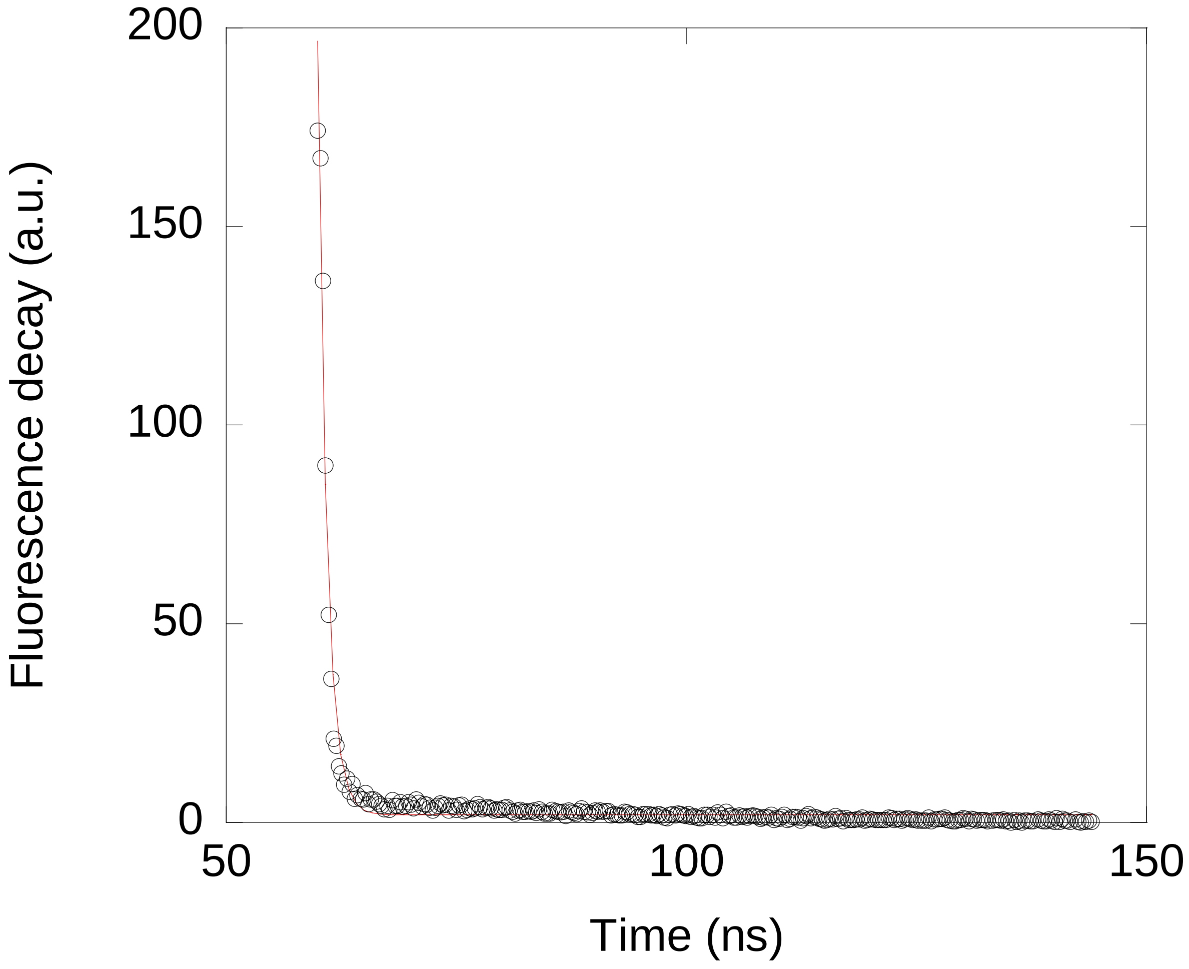

Fig. S7 (Contreras et al.)

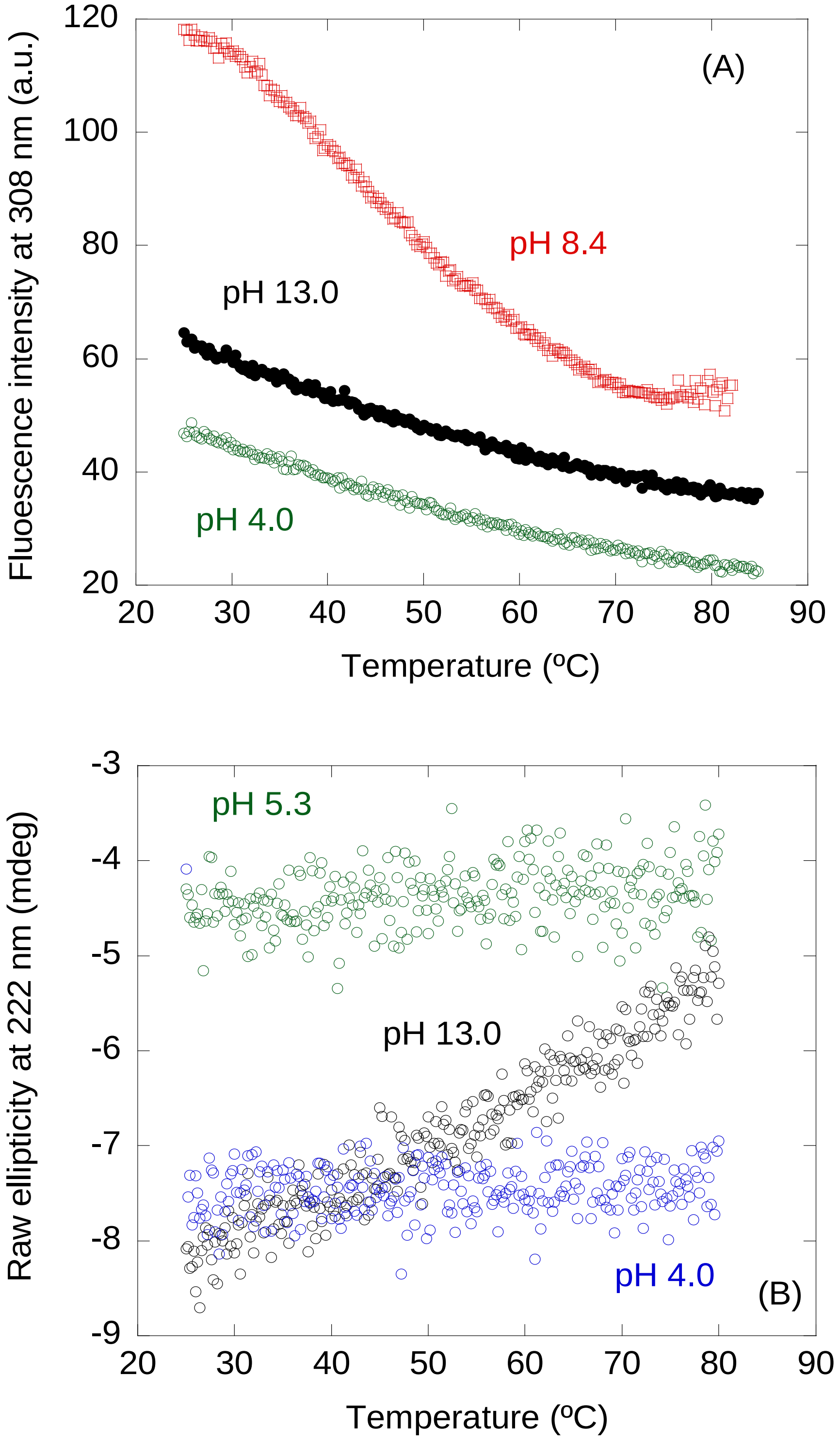

Fig. S8 (Contreras et al.)

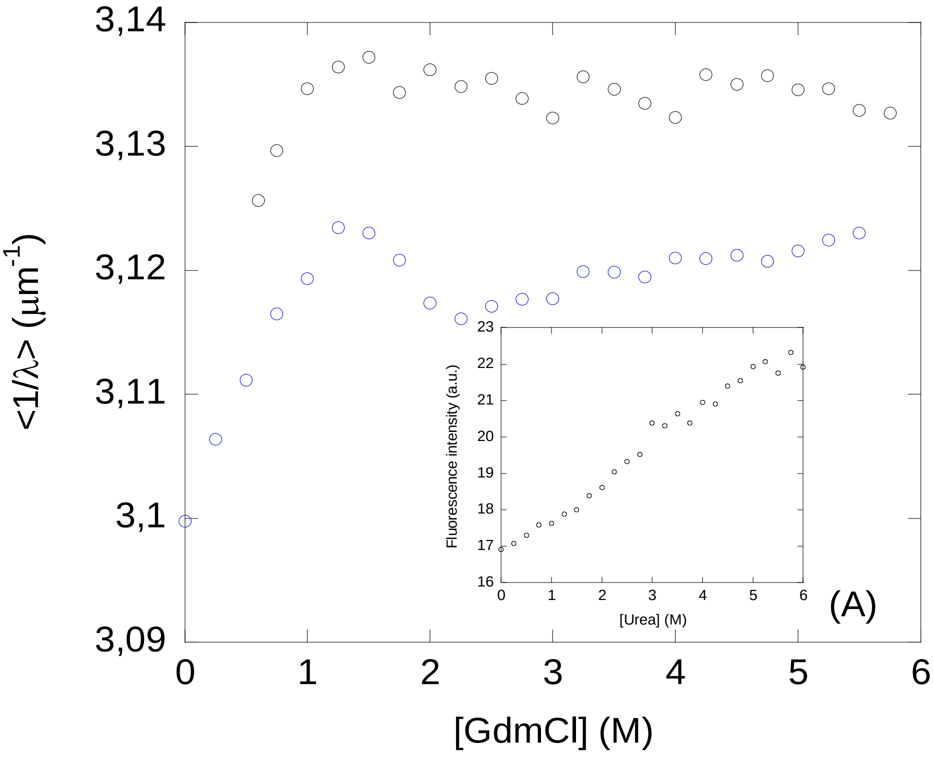
3,14
3,13
3,12
3,11
3,1
3,09
<1/λ> (μm-1)
0 1 2 3 4 5 6
[GdmCl] (M)
(A)
Fluorescence intensity (a.u.)
23 22 21 20 19 18 17 16
0 1 2 3 4 5 6
[Urea] (M)

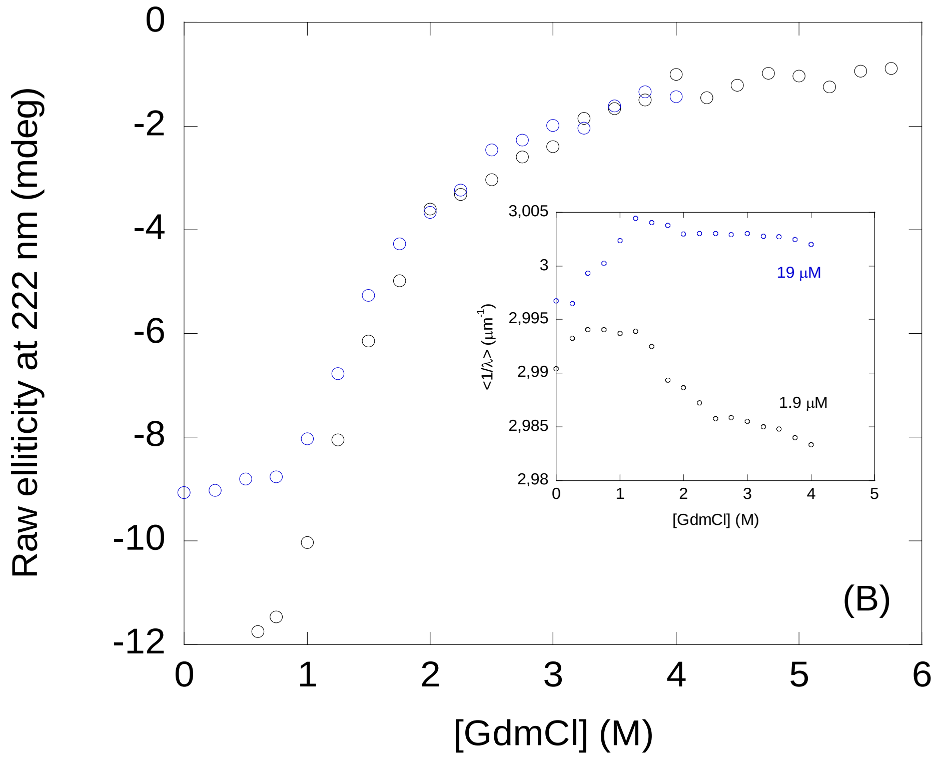
0
-2
-4
-6
-8
-10
-12
Raw ellipticity at 222 nm (mdeg)
0 1 2 3 4 5 6
[GdmCl] (M)
(B)
3,005
3
2,995
2,99
2,985
2,98
<1/λ> (μm-1)
19 μM
1.9 μM
0 1 2 3 4 5
[GdmCl] (M)

Fig. S9 (Contreras et al.)

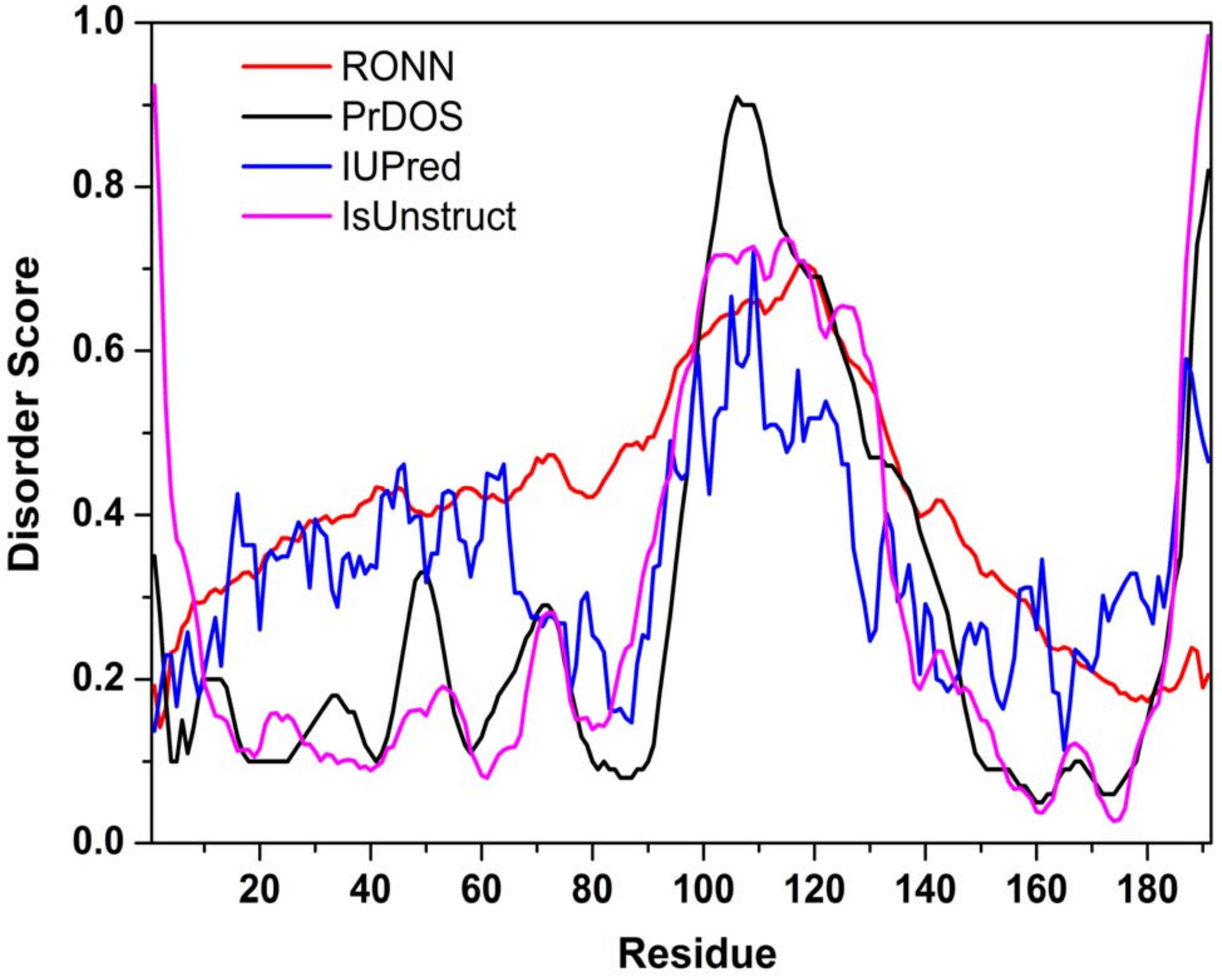

Fig. S10 (Contreras et al.)

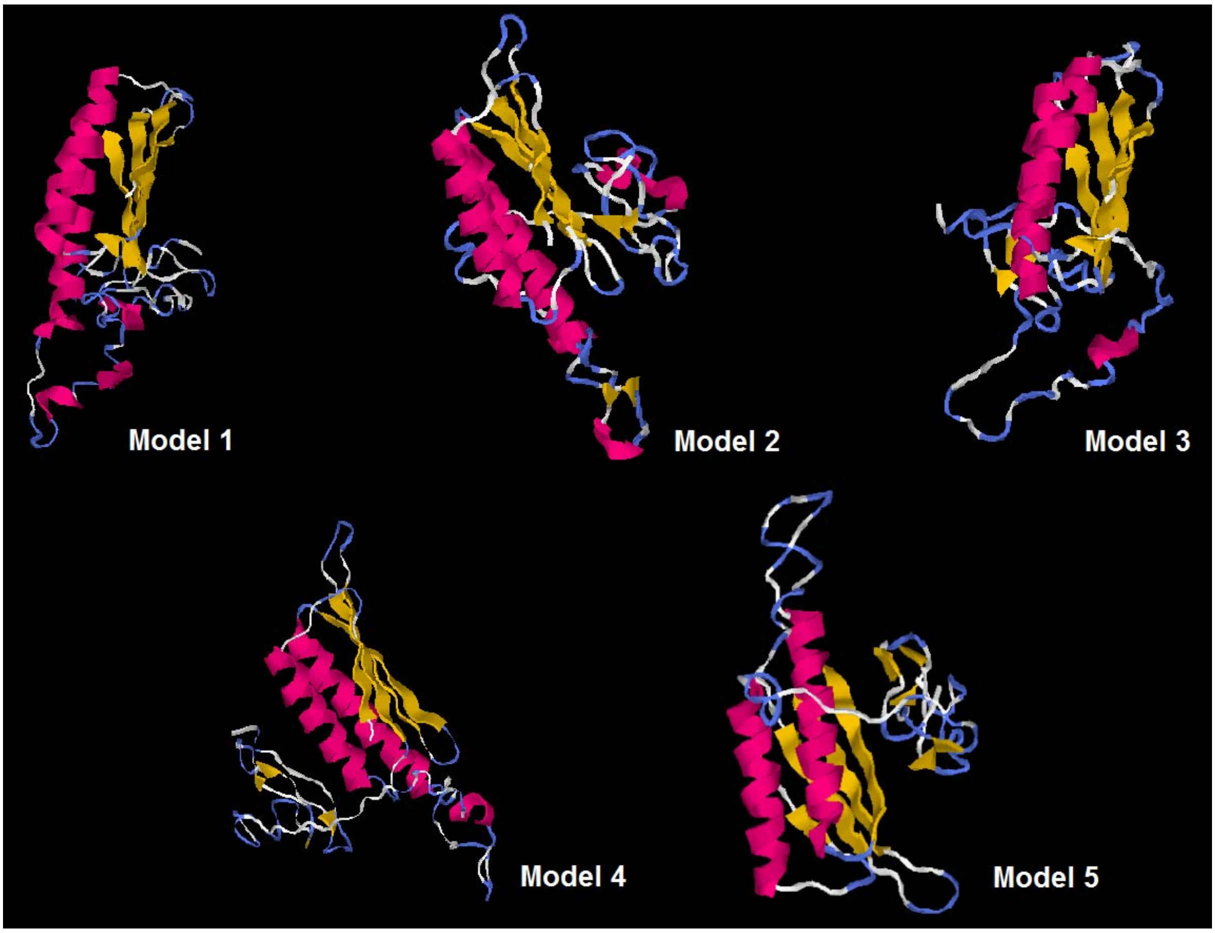

Fig. S11 (Contreras et al.)

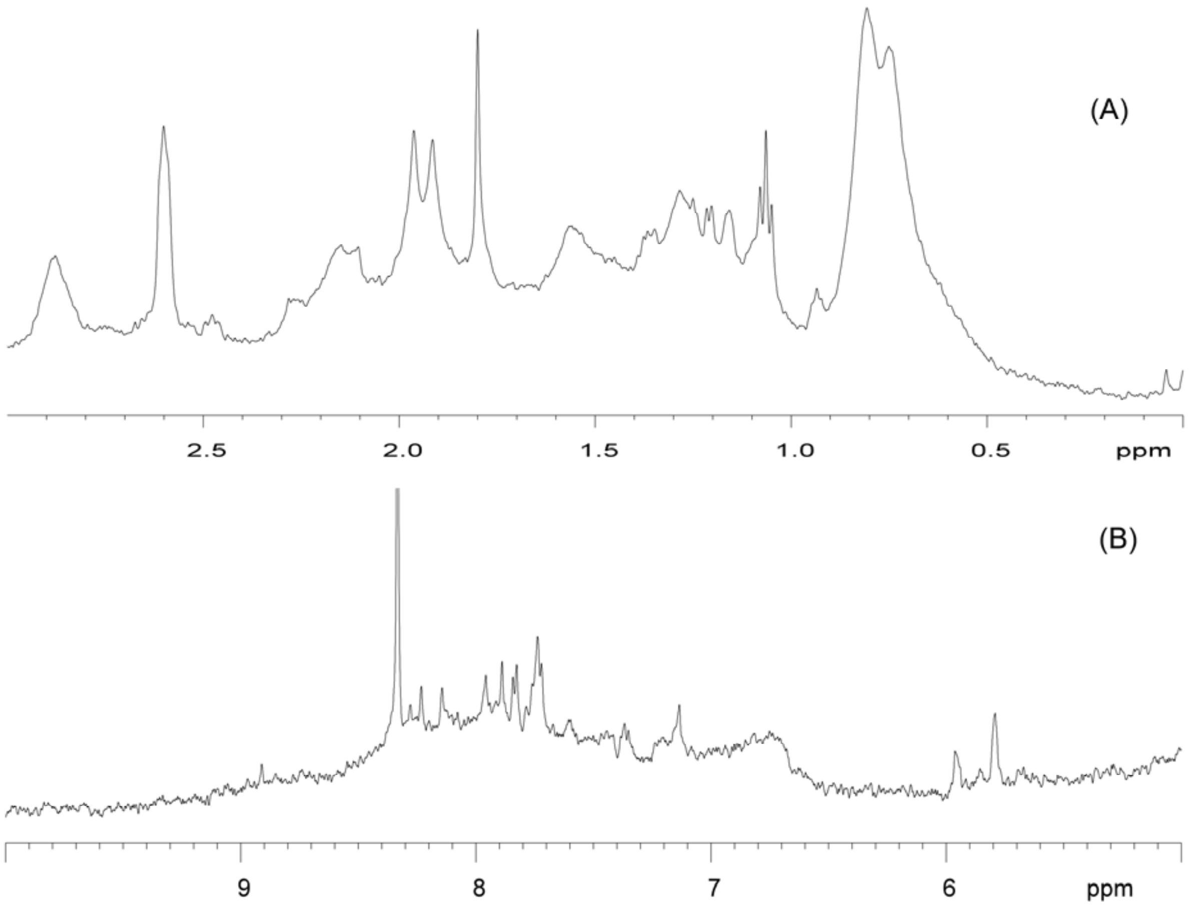

Fig. S12 (Contreras et al.)

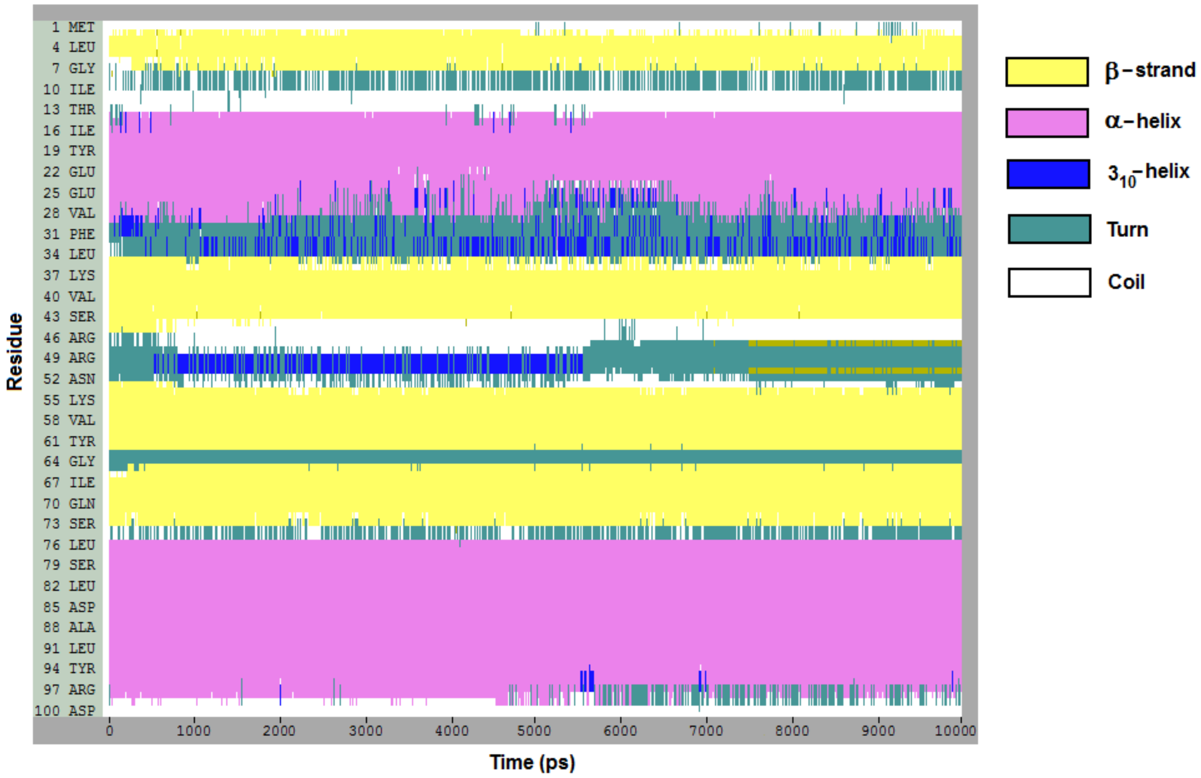

Fig. S13 (Contreras et al.)

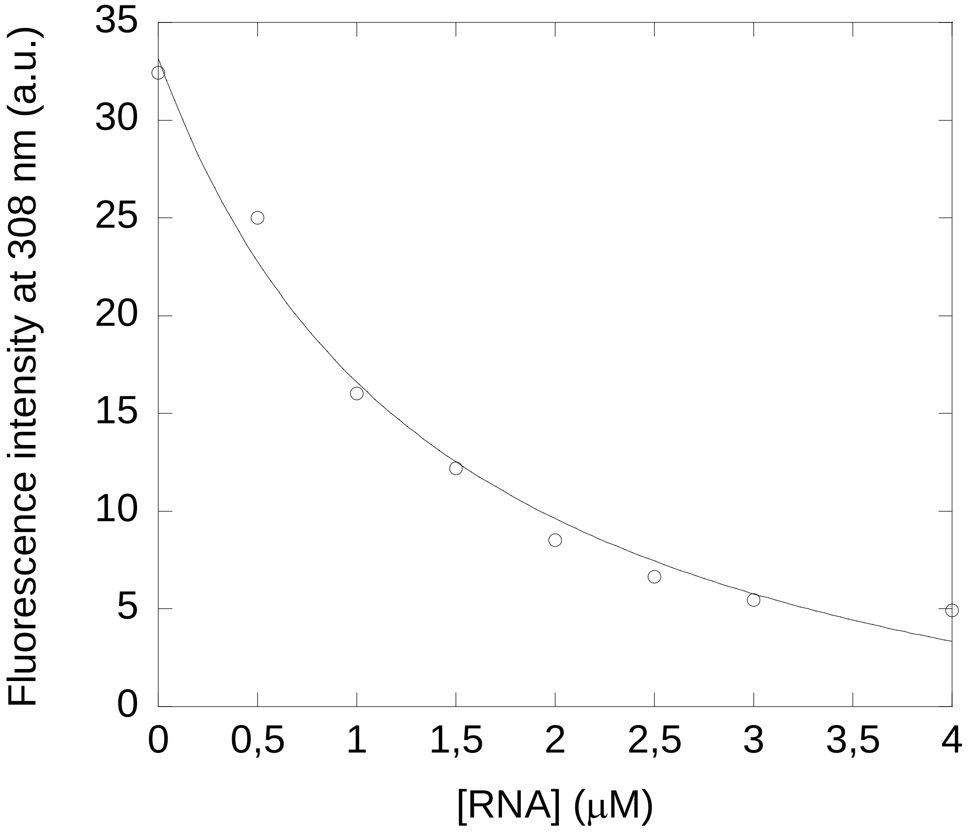

Fig. S14 (Contreras et al.)

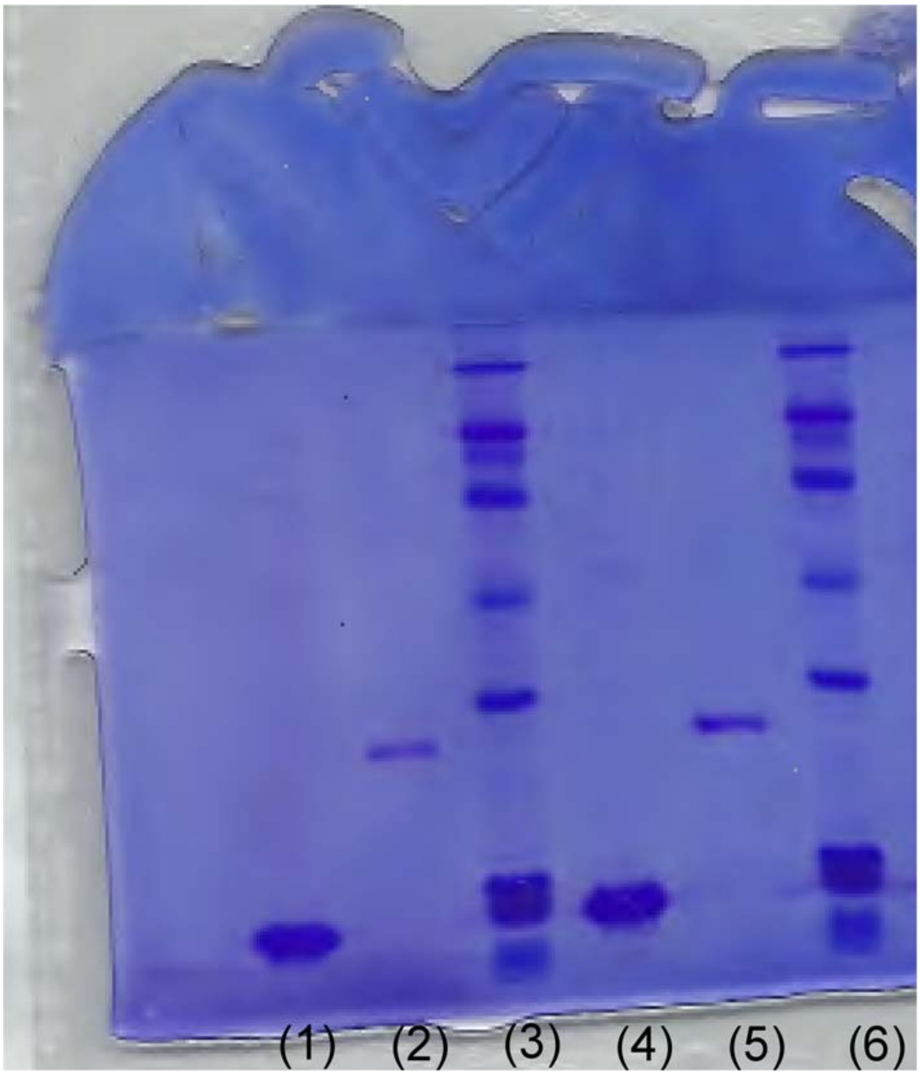

Supplement: Supplementary file 1 [file ijms-19-01857-s001.pdf]
